# Long-term mortality of Dutch COVID-19 patients admitted to the intensive care medicine: a retrospective analysis from a national quality registry

**DOI:** 10.62675/2965-2774.20240251-en

**Published:** 2024-09-18

**Authors:** Safira A. Wortel, Ferishta Bakhshi-Raiez, Ameen Abu-Hanna, Dave A. Dongelmans, Nicolette F. de Keizer, the Dutch COVID-19 Research Consortium, Aletta Houwink, Allard Dijkhuizen, Annelies Draisma, Annemiek Rijkeboer, Arjan Cloïn, Arthur de Meijer, Auke Reidinga, Barbara Festen-Spanjer, Bas van Bussel, Bob Eikemans, Cretièn Jacobs, David Moolenaar, Dharmanand Ramnarain, Dick Koning, Dirk Boer, Dirk Verbiest, Eline van Slobbe-Bijlsma, Ellen van Koppen, Els Rengers, Erik van Driel, Eva Verweij, Freya van Iersel, Gert Brunnekreef, Hans Kieft, Herman Kreeftenberg, Ilanit Hené, Inge Janssen, Ionana Drogt, Iwan van der Horst, Jan Jaap Spijkstra, Jan Rozendaal, Jannet Mehagnoul-Schipper, Jelle Epker Erasmus, Jessica Holtkamp, Johan Lutisan, Jos van Oers, Judith Lens, Laura van Gulik, Lettie van den Berg, Louise Urlings-Strop, Lyuba Georgieva, Maarten van Lieshout, Marga Hoogendoorn, Marissa Vrolijk-de Mos, Mart de Graaff, Martha de Bruin, Martijn Hoeksema, Martijn van Tellingen, Michel Barnas, Michiel Erkamp, Niels Gritters, Nuray Kusadasi, Paul Elbers, Peter Koetsier, Peter Spronk, Peter van der Voort, Ralph Pruijsten, Remko de Jong, Robert-Jan Bosman, Ronald Wesselink, Ronny Schnabel, Roy van den Berg, Ruud de Waal, Sesmu Arbous, Silvia Knape, Stefaan Hendriks, Tim Frenzel, Tom Dormans, Tom Rijpstra, Vera Silderhuis, Wouter de Ruijter

**Affiliations:** 1 University of Amsterdam Amsterdam University Medical Center Department of Medical Informatics Amsterdam The Netherlands Department of Medical Informatics, Amsterdam University Medical Center, University of Amsterdam - Amsterdam, The Netherlands.; 2 National Intensive Care Evaluation Amsterdam The Netherlands National Intensive Care Evaluation (NICE) Foundation - Amsterdam, The Netherlands.; 3 University of Amsterdam Amsterdam University Medical Center Department of Intensive Care Medicine Amsterdam The Netherlands Department of Intensive Care Medicine, Amsterdam University Medical Center, University of Amsterdam - Amsterdam, The Netherlands.; 4 Het Antoni van Leeuwenhoek Amsterdam The Netherlands Het Antoni van Leeuwenhoek - Amsterdam, The Netherlands,; 5 Rijnstate Arnhem The Netherlands Rijnstate - Arnhem, The Netherlands,; 6 Groene Hart Ziekenhuis Gouda The Netherlands Groene Hart Ziekenhuis - Gouda, The Netherlands,; 7 Flevoziekenhuis Almere The Netherlands Flevoziekenhuis - Almere, The Netherlands,; 8 Laurentius Ziekenhuis Roermond Roemond The Netherlands Laurentius Ziekenhuis Roermond - Roemond, The Netherlands,; 9 Saxenburgh Medisch Centrum Hardenberg The Netherlands Saxenburgh Medisch Centrum - Hardenberg, The Netherlands,; 10 Martini Ziekenhuis Groningen The Netherlands Martini Ziekenhuis - Groningen, The Netherlands,; 11 Ziekenhuis Gelderse Vallei Ede The Netherlands Ziekenhuis Gelderse Vallei - Ede, The Netherlands,; 12 Maastricht University Medical Center Maastricht The Netherlands Maastricht University Medical Center - Maastricht, The Netherlands,; 13 Het Van Weel-Bethesda Ziekenhuis Dirksland The Netherlands Het Van Weel-Bethesda Ziekenhuis - Dirksland, The Netherlands,; 14 Elkerliek Ziekenhuis Helmond The Netherlands Elkerliek Ziekenhuis - Helmond, The Netherlands,; 15 Martini Ziekenhuis Groningen The Netherlands Martini Ziekenhuis - Groningen, The Netherlands,; 16 Elisabeth-TweeSteden Ziekenhuis Tilburg The Netherlands Elisabeth-TweeSteden Ziekenhuis - Tilburg, The Netherlands,; 17 Catharina Ziekenhuis Eindhoven The Netherlands Catharina Ziekenhuis - Eindhoven, The Netherlands,; 18 Maasstad Ziekenhuis Rotterdam The Netherlands Maasstad Ziekenhuis - Rotterdam, The Netherlands,; 19 Admiraal De Ruyter Ziekenhuis Goes The Netherlands Admiraal De Ruyter Ziekenhuis - Goes, The Netherlands,; 20 Tergooi MC Hiversum The Netherlands Tergooi MC - Hiversum, The Netherlands,; 21 Haaglanden Medisch Centrum Den Haag The Netherlands Haaglanden Medisch Centrum - Den Haag, The Netherlands,; 22 Canisius Wilhelmina Ziekenhuis Nijmegen The Netherlands Canisius Wilhelmina Ziekenhuis - Nijmegen, The Netherlands,; 23 Alrijne Ziekenhuis Leiden The Netherlands Alrijne Ziekenhuis - Leiden, The Netherlands,; 24 Bernhoven Uden The Netherlands Bernhoven - Uden, The Netherlands,; 25 Bravis Ziekenhuis Roosendaal The Netherlands Bravis Ziekenhuis - Roosendaal, The Netherlands,; 26 ZGT Hengelo The Netherlands ZGT - Hengelo, The Netherlands,; 27 Isala Zwolle The Netherlands Isala - Zwolle, The Netherlands,; 28 St. Anna Ziekenhuis Eindhoven The Netherlands St. Anna Ziekenhuis - Eindhoven, The Netherlands,; 29 Rode Kruis Ziekenhuis Beverwijk The Netherlands Rode Kruis Ziekenhuis - Beverwijk, The Netherlands,; 30 Maasziekenhuis Pantein Beugen The Netherlands Maasziekenhuis Pantein - Beugen, The Netherlands,; 31 Ziekenhuis Nij Smellinghe Drachten The Netherlands Ziekenhuis Nij Smellinghe - Drachten, The Netherlands,; 32 Maastricht University Medical Center Maastricht The Netherlands Maastricht University Medical Center - Maastricht, The Netherlands,; 33 Amsterdam University Medical Centers Amsterdam The Netherlands Amsterdam University Medical Centers - Amsterdam, The Netherlands,; 34 Jeroen Bosch Ziekenhuis s-Hertogenbosch The Netherlands Jeroen Bosch Ziekenhuis - s-Hertogenbosch, The Netherlands,; 35 Viecuri Medisch Centrum Venlo The Netherlands Viecuri Medisch Centrum - Venlo, The Netherlands,; 36 University Medical Center Rotterdam The Netherlands University Medical Center - Rotterdam, The Netherlands,; 37 St. Jans Gasthuis Weert Weert The Netherlands St. Jans Gasthuis Weert - Weert, The Netherlands,; 38 Wilhelmina Ziekenhuis Assen Assen The Netherlands Wilhelmina Ziekenhuis Assen - Assen, The Netherlands,; 39 ZorgSaam Ziekenhuis Terneuzen The Netherlands ZorgSaam Ziekenhuis - Terneuzen, The Netherlands,; 40 IJsselland Ziekenhuis Capelle aan den IJssel The Netherlands IJsselland Ziekenhuis - Capelle aan den IJssel, The Netherlands,; 41 Meander Medisch Centrum Amersfoort The Netherlands Meander Medisch Centrum - Amersfoort, The Netherlands,; 42 HagaZiekenhuis Den Haag The Netherlands HagaZiekenhuis - Den Haag, The Netherlands,; 43 Reinier de Graaf Gasthuis Delft The Netherlands Reinier de Graaf Gasthuis - Delft, The Netherlands,; 44 Beatrixziekenhuis Gorinchem The Netherlands Beatrixziekenhuis - Gorinchem, The Netherlands,; 45 Ziekenhuis Rivierenland Tiel The Netherlands Ziekenhuis Rivierenland - Tiel, The Netherlands,; 46 Isala Zwolle The Netherlands Isala - Zwolle, The Netherlands,; 47 Langeland Ziekenhuis Zoetermeer The Netherlands Langeland Ziekenhuis - Zoetermeer, The Netherlands,; 48 St. Antonius Ziekenhuis Utrecht The Netherlands St. Antonius Ziekenhuis - Utrecht, The Netherlands,; 49 Franciscus Gasthuis & Vlietland Rotterdam The Netherlands Franciscus Gasthuis & Vlietland - Rotterdam, The Netherlands,; 50 Zaans Medisch Centrum Zaandam The Netherlands Zaans Medisch Centrum - Zaandam, The Netherlands,; 51 Ziekenhuis Tjongerschans Heerenveen The Netherlands Ziekenhuis Tjongerschans - Heerenveen, The Netherlands,; 52 Ziekenhuis Amstelland Amstelveen The Netherlands Ziekenhuis Amstelland - Amstelveen, The Netherlands,; 53 Dijklander Ziekenhuis Purmerend The Netherlands Dijklander Ziekenhuis - Purmerend, The Netherlands,; 54 Treant Zorggroep Emmen The Netherlands Treant Zorggroep - Emmen, The Netherlands,; 55 University Medical Center Utrecht Utrecht The Netherlands University Medical Center Utrecht - Utrecht, The Netherlands,; 56 Amsterdam University Medical Centers Amsterdam The Netherlands Amsterdam University Medical Centers - Amsterdam, The Netherlands,; 57 Medisch Centrum Leeuwarden Leeuwarden The Netherlands Medisch Centrum Leeuwarden - Leeuwarden, The Netherlands,; 58 Gelre Ziekenhuizen Apeldoorn The Netherlands Gelre Ziekenhuizen - Apeldoorn, The Netherlands,; 59 University Medical Center Groningen Groningen The Netherlands University Medical Center Groningen - Groningen, The Netherlands,; 60 Ikazia Ziekenhuis Rotterdam The Netherlands Ikazia Ziekenhuis - Rotterdam, The Netherlands,; 61 BovenIJ Amsterdam The Netherlands BovenIJ - Amsterdam, The Netherlands,; 62 OLVG Amsterdam The Netherlands OLVG - Amsterdam, The Netherlands,; 63 St. Antonius Ziekenhuis Utrecht The Netherlands St. Antonius Ziekenhuis - Utrecht, The Netherlands,; 64 Maastricht University Medical Center Maastricht The Netherlands Maastricht University Medical Center - Maastricht, The Netherlands,; 65 Elisabeth-TweeSteden Ziekenhuis Tilburg The Netherlands Elisabeth-TweeSteden Ziekenhuis - Tilburg, The Netherlands,; 66 Amphia Ziekenhuis Breda The Netherlands Amphia Ziekenhuis - Breda, The Netherlands,; 67 Leids Universitair Medisch Centrum Leiden The Netherlands Leids Universitair Medisch Centrum - Leiden, The Netherlands,; 68 Streekziekenhuis Koningin Beatrix Winterswijk Winterswijk The Netherlands Streekziekenhuis Koningin Beatrix Winterswijk - Winterswijk, The Netherlands,; 69 Albert Schweitzer Ziekenhuis Dordrecht The Netherlands Albert Schweitzer Ziekenhuis - Dordrecht, The Netherlands,; 70 Radboud University Medical Center Nijmegen The Netherlands Radboud University Medical Center - Nijmegen, The Netherlands,; 71 Zuyderland Medisch Centrum Heerlen The Netherlands Zuyderland Medisch Centrum - Heerlen, The Netherlands,; 72 Amphia Ziekenhuis Breda The Netherlands Amphia Ziekenhuis - Breda, The Netherlands,; 73 Medisch Spectrum Twente Enschede The Netherlands Medisch Spectrum Twente - Enschede, The Netherlands,; 74 Noordwest Ziekenhuisgroep Alkmaar The Netherlands Noordwest Ziekenhuisgroep - Alkmaar, The Netherlands.

**Keywords:** COVID-19, SARS-CoV-2, Coronavirus infections, Length of stay, Patient discharge, Survivors, Respiration, artificial, Hospital mortality, Outcome assessment, health care, Critical care, Intensive care units, Databases, factual

## Abstract

**Objective:**

To describe the 12-month mortality of Dutch COVID-19 intensive care unit patients, the total COVID-19 population and various subgroups on the basis of the number of comorbidities, age, sex, mechanical ventilation, and vasoactive medication use.

**Methods:**

We included all patients admitted with COVID-19 between March 1, 2020, and March 29, 2022, from the Dutch National Intensive Care (NICE) database. The crude 12-month mortality rate is presented via Kaplan-Meier survival curves for each patient subgroup. We used Cox regression models to analyze the effects of patient characteristics on 12-month mortality after hospital discharge.

**Results:**

We included 16,605 COVID-19 patients. The in-hospital mortality rate was 28.1%, and the 12-month mortality rate after intensive care unit admission was 29.8%. Among hospital survivors, 12-month mortality after hospital discharge was 2.5% (300/11,931). The hazard of death at 12 months after hospital discharge was greater in patients between 60 and 79 years (HR 4.74; 95%CI 2.23 - 10.06) and ≥ 80 years (HR 22.77; 95%CI 9.91 - 52.28) than in patients < 40 years of age; in male patients than in female patients (HR 1.38; 95%CI 1.07 - 1.78); and in patients with one (adjusted HR 1.95; 95%CI 1.5 - 2.53), two (adjusted HR 4.49; 95%CI 3.27 - 6.16) or more than two comorbidities (adjusted HR 4.99; 95%CI 2.62 - 9.5) than in patients with no comorbidities. Neither vasoactive medication use nor mechanical ventilation resulted in statistically significant results.

**Conclusion:**

For Dutch COVID-19 intensive care unit patients, most deaths occurred during their hospital stay. For hospital survivors, the crude 12-month mortality rate was low. Patient age (older than 60), sex and the number of comorbidities were associated with a greater hazard of death at 12 months after hospital discharge, whereas mechanical ventilation and vasoactive medication were not.

## INTRODUCTION

Since the outbreak of the coronavirus disease 2019 (COVID-19) pandemic, many studies on this disease have been published, with a particular focus on patient characteristics, treatment, and in-hospital mortality. In the Netherlands, the mean in-hospital mortality rate of COVID-19 patients in 2020 was 31%.^( 1 )^ However, different in-hospital mortality rates among intensive care unit (ICU) COVID-19 patients across different countries have been reported, ranging from 25% in a French nationwide study^( 2 )^ and 33% for a German study population^( 3 )^ to 59% for Brazilian ICU patients.^( 4 )^ This might be partly explained by differences in the organization of health care, availability of ICU beds, applied triage at ICU admission, and hospital discharge policies. To rule out differences in hospital discharge policies, analyzing mortality at a fixed moment after hospital discharge is a possible solution. Although in-hospital mortality is important and provides valuable insight, the true goal of ICU care is favorable long-term patient outcomes. In some critically ill patient groups, mortality after ICU or hospital discharge is (almost) comparable to that of the general Dutch population. These include elective and cardiac surgical patients,^( 5 )^ patients with acute intoxication^( 6 )^ and relatively young and healthy patients.^( 7 – 9 )^ In some other critically ill patient groups, such as sepsis patients and (older) pneumonia patients, long-term mortality rates can be substantial.^( 10 )^ In Sweden, a one-year mortality rate of 45% after ICU admission was reported for patients with sepsis or severe septic shock,^( 10 )^ whereas mortality rates between 19% and 51%^( 1 , 11 )^ have been reported for pneumonia patients.

We aimed to describe the long-term mortality of Dutch COVID-19 patients admitted to the ICU. Given that preexisting comorbidities and other patient characteristics can markedly influence the long-term outcome of patients, the analyses were performed both in the total group of COVID-19 patients as well as in various subgroups of COVID-19 patients (based on the number of comorbidities before hospitalization, age, sex, and mechanical ventilation [MV] and the use of vasoactive medication in the first 24 hours of ICU admission) adjusted for confounders. Finally, we compared the 12-month mortality of the ICU COVID-19 population that survived hospital admission to the mortality of the general Dutch population.

## METHODS

### Data source

We retrospectively collected data from the Dutch National Intensive Care Evaluation (NICE) registry. The NICE registry is a national quality registry established in 1996. Since 2016, all Dutch ICUs have participated in the NICE registry and regularly send data to the registry. In turn, the NICE registry provides the participating ICUs with benchmark reports and access to an online tool enabling each ICU to analyze their own data for room for improvement.^( 12 , 13 )^ The NICE registry database contains, among other data, demographic, physiological, clinical, ICU, and in-hospital mortality data for all COVID-19 patients admitted to Dutch ICUs.^( 12 , 13 )^ Patients were considered to have COVID-19 if the reverse transcription polymerase chain reaction (RT-PCR) of their respiratory secretions was positive for severe acute respiratory syndrome coronavirus 2 (SARS-CoV-2) or if their CT scan was consistent with COVID-19, i.e., if the COVID-19 Reporting and Data System (CO-RADS) score was ≥ 4 in combination with the absence of an alternative diagnosis.^( 14 )^ The date of death after hospital discharge was derived for each patient by linking patient data from the NICE registry to ‘Vektis’ data, a national administrative claims database of health insurance companies.^( 15 )^

### Study outcomes

We included all patients admitted with COVID-19 between March 1, 2020, and March 29, 2022, from the Dutch National Intensive Care (NICE) database. We used two starting points to determine the follow-up period: the first was the ICU admission date, and the second was the hospital discharge date. With the Vektis data, mortality up to 12 months after ICU admission and after hospital discharge could be determined.

Surgical patients and patients with missing key data, such as hospital discharge date and hospital discharge status, were excluded from the analyses. Patients with missing body mass index (BMI) data were excluded from the models that included BMI as a covariate. Finally, the expected 12-month mortality rate for the general Dutch population was assessed by using sex- and age-specific mortality rates reported by the Dutch governmental institution Statistics Netherlands, i.e., ‘CBS’.^( 16 )^

### Statistical analyses

Patient baseline characteristics and outcomes are described via descriptive statistics. Continuous variables are presented in terms of the means and standard deviations (SD) or medians and interquartile ranges (IQR) depending on their distributions, whereas categorical variables are presented as the proportions. Baseline characteristics are reported for hospital nonsurvivors and for survivors. Crude long-term mortality is presented and analyzed via Kaplan-Meier survival curves and the log-rank test to compare the curves. Patient characteristics and Kaplan-Meier curves are shown for the whole study population and for various patient subgroups. These subgroups were based on the patients’ age, sex, number of comorbidities, and MV and vasoactive medication use in the first 24 hours of ICU admission. To categorize patients into age groups, the following groups were defined: patients younger than 40 years, 40 - 59 years, 60 - 79 years, and 80 years and older. To categorize COVID-19 patients on the basis of the number of comorbidities (none, one, two, or more than two), the following comorbidities available in the NICE registry were considered: immunological insufficiency, chronic renal failure, chronic respiratory insufficiency, chronic cardiovascular insufficiency, cirrhosis, malignancy, and diabetes. To categorize patients by MV, we considered any instance of MV occurring within the initial 24 hours of admission. No further distinction could be made between invasive and noninvasive MV. Intravenous vasoactive medication use includes positive inotropes, such as vasopressin, dopamine, dobutamine hydrochloride, vasopressors and phosphodiesterase inhibitors, in the first 24 hours of ICU admission.

We used Cox regression models to analyze the effects of the characteristics of patients who survived hospital admission on mortality during the 12 months after hospital discharge. In each multivariate model, we adjusted for covariates on the basis of a directed cyclic graph (DAG)^( 17 )^ using clinical knowledge. We also used Cox regression models with an additional adjustment for Acute Physiology and Chronic Health Evaluation (APACHE) IV mortality probability as a sensitivity analysis because of the associations between the APACHE IV mortality probability and various confounders and/or prognostic factors. For this sensitivity analysis, we thus excluded patients for whom the APACHE IV exclusion criteria were applicable.^( 18 )^ These criteria include patients with an ICU length-of-stay of less than 4 hours or longer than one year, patients who are readmitted, patients with a missing admission diagnosis or admission type, burn patients, organ transplantation patients, and coronary care unit or recovery patients.

According to the NICE data definitions, a recovery patient is a patient admitted to the ICU only under the responsibility of the anesthesiologist and immediately following an operation whereby the duration of treatment in the ICU does not exceed 4 hours. Finally, we calculated the proportions of men and women in each age group. The weighted average of the mortality rates of the general Dutch population was assessed according to the proportion of patients in each group. We compared the weighted average 12-month mortality rate of the general Dutch population with the 12-month mortality rate of the Dutch COVID-19 ICU population.

**Figure 1:**
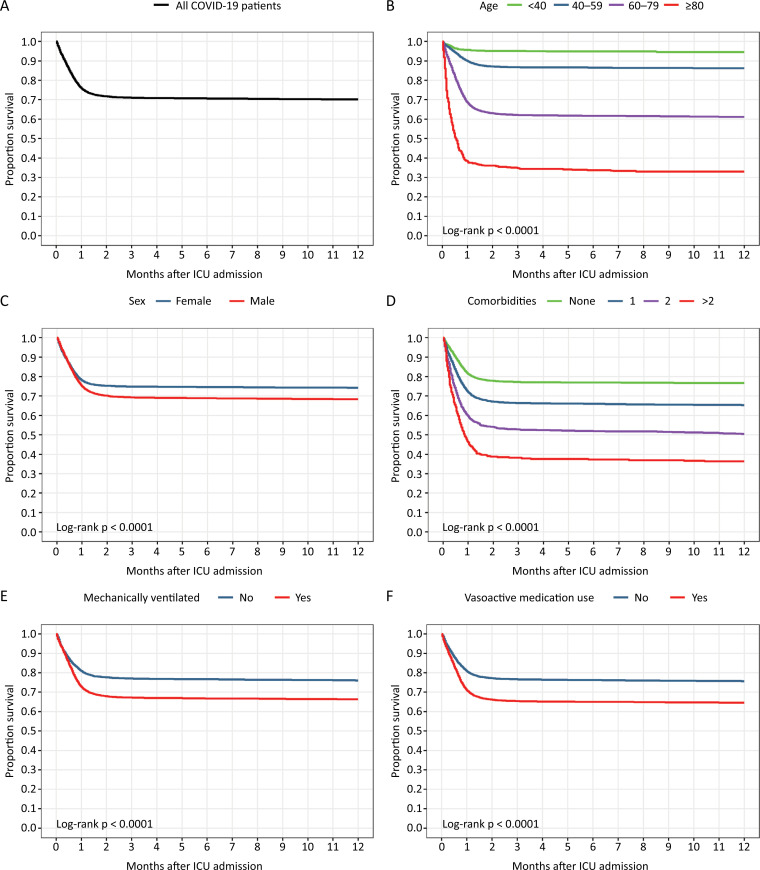
Kaplan-Meier survival curves of COVID-19 patients after intensive care unit admission.

All analyses were performed via the R statistical environment (version 4.2.0), and the DAGs were drawn via DAGitty version 3.0^( 19 )^ ( Figures 1SA - 1SE - Supplementary Material ).

The Medical Ethics Review Committee of the Academic Medical Center Amsterdam reviewed the research proposal and waived the need for informed consent (reference number W21_091 # 21.102).

## RESULTS

In total, 17,686 COVID-19 ICU patients were admitted to a Dutch ICU from March 1, 2020, to March 29, 2022. After excluding surgical patients and patients with missing discharge data or missing hospital discharge data (6.1%), we included 16,605 patients in our analyses. Table 1 shows the characteristics of the included patients. The median age at admission was 64 years; 68.4% of the included patients were male, and 61% of patients were mechanically ventilated in the first 24 hours of ICU admission. In the first 24 hours after ICU admission, 49.9% of the patients received vasoactive medication. The most prevalent chronic comorbidity was diabetes (22.3%), followed by immunological insufficiency (9.8%) and chronic respiratory insufficiency (4.3%). The median APACHE IV mortality probability was 0.22.

**Table 1:** Characteristics of COVID-19 patients who could be followed for 12 months after intensive care unit admission

		All included ICU patients	Hospital survivors	Hospital nonsurvivors
Number of patients		16,605	11,931	4,674
Age		64 (55 - 71)	61 (52 - 69)	70 (63 - 74)
	< 40	1,006 (6.1)	958 (8)	48 (1)
	40 - 59	5,154 (31)	4,492 (37.6)	662 (14.2)
	60 - 79	9,979 (60.1)	6,304 (52.8)	3,675 (78.6)
	≥ 80	466 (2.8)	177 (1.5)	289 (6.2)
Sex, male		11,351 (68.4)	7,959 (66.7)	3,392 (72.6)
BMI (kg/m^2^)		28.7 (25.7 - 32.5)	29 (25.9 - 32.8)	28 (25.2 - 31.8)
	< 18.5	97 (0.6)	67 (0.6)	30 (0.7)
	18.5 - 24.9	3,191 (19.6)	2,137 (18.2)	1,054 (23.1)
	25 - 29.9	6,492 (39.8)	4,601 (39.3)	1,891 (41.4)
	30 - 34.9	3,999 (24.5)	2,996 (25.6)	1,003 (21.9)
	35 - 39.9	1,656 (10.2)	1,254 (10.7)	402 (8.8)
	≥ 40	857 (5.3)	665 (5.7)	192 (4.2)
Malignancy		447 (2.7)	213 (1.8)	234 (5)
Immunological insufficiency		1,634 (9.8)	929 (7.8)	705 (15.1)
Chronic respiratory insufficiency		712 (4.3)	438 (3.7)	274 (5.9)
Chronic renal failure		707 (4.3)	319 (2.7)	388 (8.3)
Chronic cardiovascular insufficiency		257 (1.5)	130 (1.1)	127 (2.7)
Cirrhosis		83 (0.5)	38 (0.3)	45 (1)
Diabetes		3,710 (22.3)	2,417 (20.3)	1,293 (27.7)
Number of comorbidities				
	0	9,836 (59.2)	7,658 (64.2)	2,178 (46.6)
	1	4,974 (30)	3,351 (28.1)	1,623 (34.7)
	2	1,470 (8.9)	795 (6.7)	675 (14.4)
	> 2	325 (2)	127 (1.1)	198 (4.2)
MV in first 24 hours of ICU admission		10,128 (61)	6,869 (57.6)	3,259 (69.7)
Vasoactive medication in first 24 hours of ICU admission		8,282 (49.9)	5,475 (45.9)	2,807 (60.1)
APACHE III APS		48 (39 - 58)	46 (37 - 55)	54 (44 - 67)
APACHE IV mortality probability		0.22 (0.14 - 0.34)	0.19 (0.12 - 0.29)	0.32 (0.22 - 0.48)

ICU - intensive care unit; BMI - body mass index; MV - mechanical ventilation; APACHE - Acute Physiology and Chronic Health Evaluation; APS - acute physiology score. The results are expressed as n, median (interquartile range) or n (%).

Compared with the hospital nonsurvivors, the hospital survivors were younger (median age = 61), had a smaller proportion of men (66.7%) and had fewer patients with comorbidities (median age = 70, 72.6% male; Table 1 ).

Table 2 shows the patient outcomes. The median length of stay in the ICU was 11 days, and the median hospital length of stay was 20 days. Overall, 28% of the ICU patients died during hospital admission. Among the COVID-19 patients who survived hospital admission, 2.5% died within 12 months after hospital discharge ( Table 2 ).

**Table 2:** Outcomes of COVID-19 patients

	All included ICU patients
Number of patients	16,605
LOS ICU in days	11 (6 - 22)
LOS hospital in days	20 (12 - 32)
ICU mortality	4,084 (24.6)
Hospital mortality	4,674 (28.1)
12-month mortality after ICU admission	4,956 (29.8)
12-month mortality after hospital discharge *	300 (2.5)

ICU - intensive care unit; LOS - length of stay.

* Calculated for the intensive care unit patients who survived hospital admission (N = 11,931). The results are expressed as n, median (interquartile range) or n (%).

### Crude all-cause 12-month mortality

The Kaplan-Meier survival curves for COVID-19 patients in figure 1 show that mortality mainly occurred in the first two months after ICU admission. The Kaplan-Meier survival curves revealed that patients younger than 40 years had a more favorable outcome than patients in other age groups; female patients had a more favorable outcome than male patients; patients who were not mechanically ventilated had a more favorable outcome than patients who were mechanically ventilated in the first 24 hours of ICU admission; patients with no comorbidities had a more favorable outcome than patients with one, two or more comorbidities; and patients who received vasoactive medication in the first 24 hours of ICU admission had a worse outcome than patients who did not receive vasoactive medication ( Figure 1 ).

(A) All included COVID-19 patients; (B) by age group; (C) by sex; (D) by number of comorbidities; (E) by mechanical ventilation in the first 24 hours of ICU admission; (F) by vasoactive medication use in the first 24 hours of intensive care unit admission. ICU - intensive care unit.

**Figure 2:**
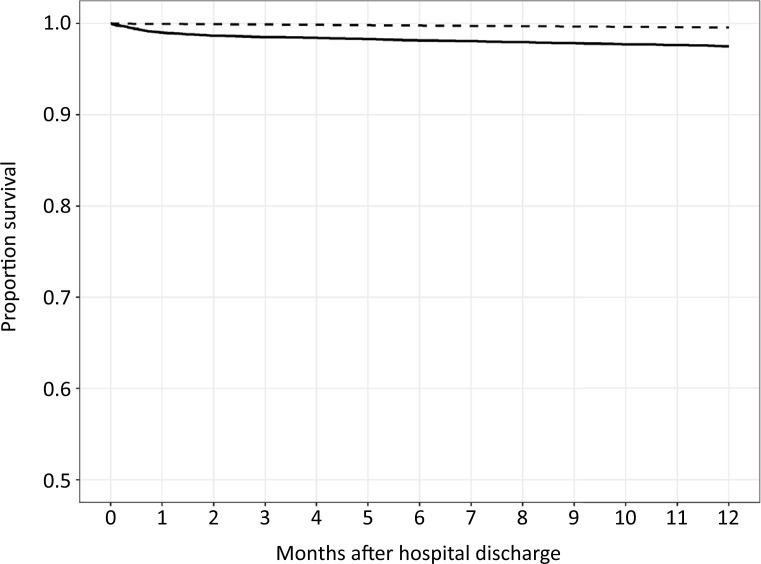
Kaplan-Meier survival curves of COVID-19 patients after hospital discharge compared with those of the general Dutch population. The solid line represents the COVID-19 intensive care unit study population, and the dashed line represents the general Dutch population.

Figure 2 shows the crude mortality rates after hospital discharge for COVID-19 patients and for the general Dutch population. The crude cumulative mortality of the COVID-19 patients at the end of the 12-month follow-up period was greater than the weighted average of the sex- and age-specific mortality rates in the general Dutch population, i.e., 2.5% and 0.4%, respectively.

On the basis of the unadjusted hazard ratios, we observed an increased hazard of death during the 12 months after hospital discharge in patients aged between 60 and 79 years (hazard ratio [HR] 4.74; 95% confidence interval [95%CI] 2.23 - 10.06) and patients aged 80 years or older (HR 22.77; 95%CI 9.91 - 52.28) compared with patients aged younger than 40 years. Furthermore, compared with female patients, male patients had an increased hazard of death at 12 months after hospital discharge (HR 1.38; 95%CI 1.07 - 1.78). Patients with one (HR 1.95; 95%CI 1.5 - 2.53), two (HR 4.49; 95%CI 3.27 - 6.16), or more than two comorbidities [HR 4.99; 95%CI 2.62 - 9.5) had an increased hazard of death during the 12 months after hospital discharge compared with patients without comorbidities ( Table 3 ). The results for MV and vasoactive medication were not statistically significant. Only the DAGs for the number of comorbidities and vasoactive medication use resulted in a confounder set for which adjustment was necessary. After adjustment for age and BMI, the number of comorbidities was still statistically significant (adjusted HR 1.69; 95%CI 1.3 - 2.2; adjusted HR 3.65; 95%CI 2.66 - 5.03; adjusted HR 4.16; 95%CI 2.18 - 7.94, for 1, 2, and > 2 comorbidities). In this model, 211 patients (1.8% of the hospital survivors) were excluded because of missing BMI data. After adjustment for MV, vasoactive medication did not yield statistically significant results (adjusted HR 1.08; 95%CI 0.8 - 1.46).

**Table 3:** Unadjusted hazard ratios for mortality in COVID-19 patients after hospital discharge

		Number of patients	HR	95%CI
Age				
	< 40	958	Reference	
	40 - 59	4,492	1.56	(0.71 - 3.43)
	60 - 79	6,304	4.74	(2.23 - 10.06)
	≥ 80	177	22.77	(9.91 - 52.28)
Sex				
	Female	3,972	Reference	
	Male	7,959	1.38	(1.07 - 1.78)
Number of comorbidities				
	0	7,523	Reference	
	1	3,293	1.95	(1.5 - 2.53)
	2	778	4.49	(3.27 - 6.16)
	> 2	126	4.99	(2.62 - 9.5)
MV in first 24 hours of ICU admission				
	No	5,062	Reference	
	Yes	6,869	0.85	(0.68 - 1.07)
Vasoactive medication in first 24 hours of ICU admission				
	No	6,456	Reference	
	Yes	5,475	0.94	(0.75 - 1.18)

HR - hazard ratio; 95%CI - 95% confidence interval; MV - mechanical ventilation; ICU - intensive care unit.

In the sensitivity analysis, we included hospital survivors for whom the APACHE IV exclusion criteria were not applicable (n = 11,702). The unadjusted HRs were similar to those of the main analyses both in size and statistical significance ( Table 3 and Table 1S - Supplementary Material ). After adjustment for the APACHE IV mortality probability, we found that the HRs for age, sex, number of comorbidities and MV were significantly associated with 12-month mortality after hospital discharge ( Table 1S - Supplementary Material ). For vasoactive medication use, the adjusted HR was still not significantly associated with 12-month mortality.

The magnitude of the HRs lower than 1 in the Cox models for MV, as shown in table 3 and the table 1S (Supplementary Material) , was surprising. This prompted us to perform additional analyses. We compared mechanically ventilated patients with nonmechanically ventilated patients with COVID-19. The patient characteristics between the two groups were comparable ( Table 2S - Supplementary Material ). In another analysis, we excluded patients admitted to the ICU during the first peak of the COVID-19 pandemic (ICU admission date before May 24, 2020) because oxygen therapy changed after that period. This additional analysis showed results in the same direction: an HR of 0.92 (95%CI 0.73 - 1.17) ( Table 3S - Supplementary Material ).

## DISCUSSION

### Main findings

Mortality in Dutch COVID-19 patients admitted to the ICU was high during hospital admission (28%) and occurred mainly within the first two months after ICU admission and within the hospital. Among hospital survivors, the mortality rate was relatively low (2.5%) following the first 12 months after hospital discharge. However, when we compared the results of the hospital survivors with those of the general population, we found that the COVID-19 patient population consistently had slightly worse crude mortality outcomes. Among COVID-19 patients who survived hospital admission, those who were in older age groups (between 60 and 79 years old and 80 years or older) or had one, two or more than 2 comorbidities had a significantly greater hazard of death during the 12 months after hospital discharge than did patients younger than 40 years and patients without comorbidities. Furthermore, patients who were mechanically ventilated in the first 24 hours after ICU admission had a decreased hazard of death within 12 months after hospital discharge, but only after we adjusted for APACHE IV severity of illness. We found no statistically significant effect of vasoactive medication use on 12-month mortality after hospital discharge.

### Comparison with prior studies

In a recently published study on the long-term outcomes of COVID-19 patients, Hägglöf et al. reported that mortality beyond 90 days was strikingly low and that this indicated a high probability of survival after the acute phase of illness.^( 20 )^ They reported that men had a greater risk of one-year mortality (HR 1.13; 95%CI 1.04 - 1.24). This is consistent with our findings. Furthermore, we reported an in-hospital mortality rate of 28%, in line with other COVID-19 studies, although there is wide variation in reported in-hospital mortality rates between studies on COVID-19 ICU patients (25% - 59%).^( 2 – 4 , 21 )^ The variation in mortality might be explained by the time period in which patients were admitted to the ICU, the strain of the SARS-CoV-2 virus and the COVID-19 vaccination rates.^( 22 , 23 )^ Other COVID-19 studies, such as that of Ceccato et al., reported a crude 35% 1-year mortality rate, and 1% of discharged patients died within the first year after ICU admission.^( 24 )^ In a Swedish study, a 90-day mortality rate of 26.9% after ICU admission was reported, and the number of deaths between 90 days and the end of follow-up from March to October 2020 was low (n = 11 of 2,354 included patients).^( 25 )^ In this study, however, the follow-up period was shorter, and male sex was identified as a risk factor for mortality (HR 1.28; 95%CI 1.06 - 1.55). In our study, we also found that sex was associated with long-term mortality. Novelli et al. reported an overall one-year mortality rate of 33.6% for COVID-19 patients and a mortality rate of 3.7% for COVID-19 patients who were discharged alive from the hospital.^( 26 )^ This corresponds with our findings: we reported a 2.5% mortality rate 12 months after hospital discharge. Although they included pneumonia and acute respiratory distress syndrome patients from a single center who were admitted during the first COVID-19 peak, there were other similarities. The majority of patients died during hospital admission, and patient age over 65 years was an independent predictor of one-year postdischarge mortality. In a Danish study in which patients were followed for six months, male sex, age, and chronic comorbidities were associated with an increased risk of death.^( 27 )^

Patient characteristics associated with long-term mortality are also in line with earlier studies in the general ICU population. Atramont et al. and Szakmany et al. reported that (older) age was associated with long-term mortality.^( 8 , 28 )^ Szakmany^( 28 )^ also reported an association between the number of comorbidities and long-term outcomes. In a multivariable analysis, Gayat et al. identified age, comorbidities, red blood cell transfusion, ICU length of stay and abnormalities in common clinical factors at the time of ICU discharge (low systolic blood pressure, temperature, total protein, platelet count and white cell count) as independent factors associated with 1-year mortality.^( 7 )^ However, we could not completely compare these results with our study, as we did not collect the same variables, and multivariable logistic regression was used to determine the variables associated with 1-year mortality.

We found the magnitude of the HRs of the mechanically ventilated Cox models in table 3 and table 1S (Supplementary Material ) surprising. An HR lower than one suggests that mechanically ventilated patients have better 12-month survival than do COVID-19 patients who are not mechanically ventilated. This prompted us to perform additional analyses. When we compared mechanically ventilated patients with nonmechanically ventilated patients with COVID-19, we observed no strong differences in patient characteristics between the two groups ( Table 2S - Supplementary Material ). In another analysis, we excluded patients admitted to the ICU in the first COVID-19 peak (ICU admission date before May 24, 2020) because oxygen therapy changed during the COVID-19 pandemic. During the first peak of the COVID-19 pandemic, most COVID-19 patients were commonly treated with MV, while among COVID-19 patients admitted after the first peak of the COVID-19 pandemic, there was greater availability of high-flow nasal oxygen (HFNO) as well as experience indicating that this could be used safely. More patients were treated with HFNO and, when necessary, MV. High-flow nasal oxygen is not considered a form of MV. The additional analysis revealed results in the same direction: an HR of 0.92 (95%CI: 0.73 - 1.17) ( Table 3S - Supplementary Material ). This finding was, however, not statistically significant.

Our results revealed a reinforcing effect of the APACHE IV score because the HR of MV decreased from 0.85 to 0.68 ( Table 1S - Supplementary Material ). This finding is not surprising because mechanically ventilated patients are the sickest patients; thus, when adjusting for illness severity, it is not surprising that the effect becomes even stronger. What remains surprising is why MV seemed to have a protective effect in the long term. As in previous studies, patients in need of MV had worse long-term outcomes.^( 29 )^ When MV is not scarce, which is mostly the case in the Netherlands, we often consider MV as a patient characteristic of someone who is more severely ill than someone who is not mechanically ventilated and thus has a lower survival chance. However, especially at the beginning of the pandemic, MV was scarce. The results seem to show that, in this population, MV has a protective effect on long-term outcomes. Another explanation might be residual confounding, as care providers decide on MV on the basis of data not available to us.

On the basis of our study data, we can assume that receiving MV is beneficial in the long term compared with not receiving MV. We usually think of variable MV as more of a patient characteristic of someone who is more severely ill than someone who is not mechanically ventilated and thus has worse survival chances. However, within this studied COVID-19 population, patients did not receive MV during the first 24 hours of ICU admission and should have been mechanically ventilated because this treatment is protective in the long term. Finally, for the patients in our study who were not mechanically ventilated, they may have received MV during their ICU treatment after the first 24 hours after ICU admission. Because we do not know whether all patients received MV after the first 24 hours, it was not possible to analyze whether this had a protective effect.

### Strengths and limitations

This study is one of the few to report a long-term follow-up of 12 months for COVID-19 patients after ICU treatment. Moreover, this study included high-quality data from a national quality registry that covers all COVID-19 ICU patients in an entire country. This study also has several limitations. Compared with other Western countries, the Netherlands has relatively few ICU beds; this might hamper the generalizability of our results to other Western countries with greater ICU capacity. Compared with other contexts, such as low–middle income countries (LMICs), the results could be difficult to extrapolate. In a previous study conducted in Brazil, COVID-19 patients in need of MV had worse survival and quality of life.^( 29 )^ In addition, LMICs, such as Brazil, have more variation in staffing ratios and other organizational factors than the Netherlands.^( 30 )^ Luckily, there are initiatives such as the linking of global intensive care (LOGIC), in which national quality registries participate and research ideas are carried out to benchmark ICU performance between various countries.^( 31 )^

We used the national administrative claims database Vektis on the date of death for long-term survival analyses. If a patient did not have a date of death, this patient was considered alive at the end of the study period. This could have led to an overestimation of survivors. However, the date of death was administered in a timely and reliable manner to directly stop the collection of monthly health insurance premiums from deceased persons; thus, we believe that this likely did not greatly impact the results.

### Clinical implications and recommendations for future studies

In this study, we observed that the majority of patients died in the first two months after ICU admission and that, after hospital discharge, the number of events was low. However, COVID-19 patients still had slightly worse outcomes than the general Dutch population. We observed a small increase in mortality immediately after hospital discharge. This might indicate that patients were released for home-based end-of-life care or were discharged to an end-of-life care facility. Furthermore, in this study, we focused on the long-term survival of COVID-19 patients, but to obtain a complete understanding of the long-term consequences of COVID-19, it is also important to analyze more patient-centered long-term outcomes, such as quality of life and other patient-reported outcomes. Smaller studies have shown that, among COVID-19 patients, long COVID-19 is a challenging and substantial problem.^( 32 – 35 )^

## CONCLUSION

Among COVID-19 intensive care unit patients, the mortality rate is highest in the first two months after intensive care unit admission and then stabilizes over time. However, the crude mortality 12 months after hospital discharge is higher than that of the general Dutch population. Among the subgroups, patients older than 60 years, male patients and patients with comorbidities had significantly higher hazard rates of death at 12 months after hospital discharge than patients < 40 years, female patients and patients with no comorbidities.
