# Supplementary material for: Long-term mortality of Dutch COVID-19 patients admitted to the intensive care medicine: a retrospective analysis from a national quality registry
Source: Crit Care Sci. 2024 Sep 18;36:e202400251en. doi: 10.62675/2965-2774.20240251-en (PMC11463994; doi:10.62675/2965-2774.20240251-en)
Supplement: Supplementary file 1 [file 2965-2774-ccsci-36-e202400251en-suppl01.pdf]

Critical Care Science
https://doi.org/10.62675/2965-2774.20240251-en

SUPPLEMENTARY MATERIAL

Long-term mortality of Dutch COVID-19 patients admitted to the intensive care medicine: a retrospective analysis from a national quality registry

Safira A. Wortel[1,2,3], Ferishta Bakhshi-Raiez[1,2,3], Ameen Abu-Hanna[1,2], Dave A. Dongelmans[2,3,4], Nicolette F. de Keizer[1,2,3], the Dutch COVID-19 Research Consortium[5]

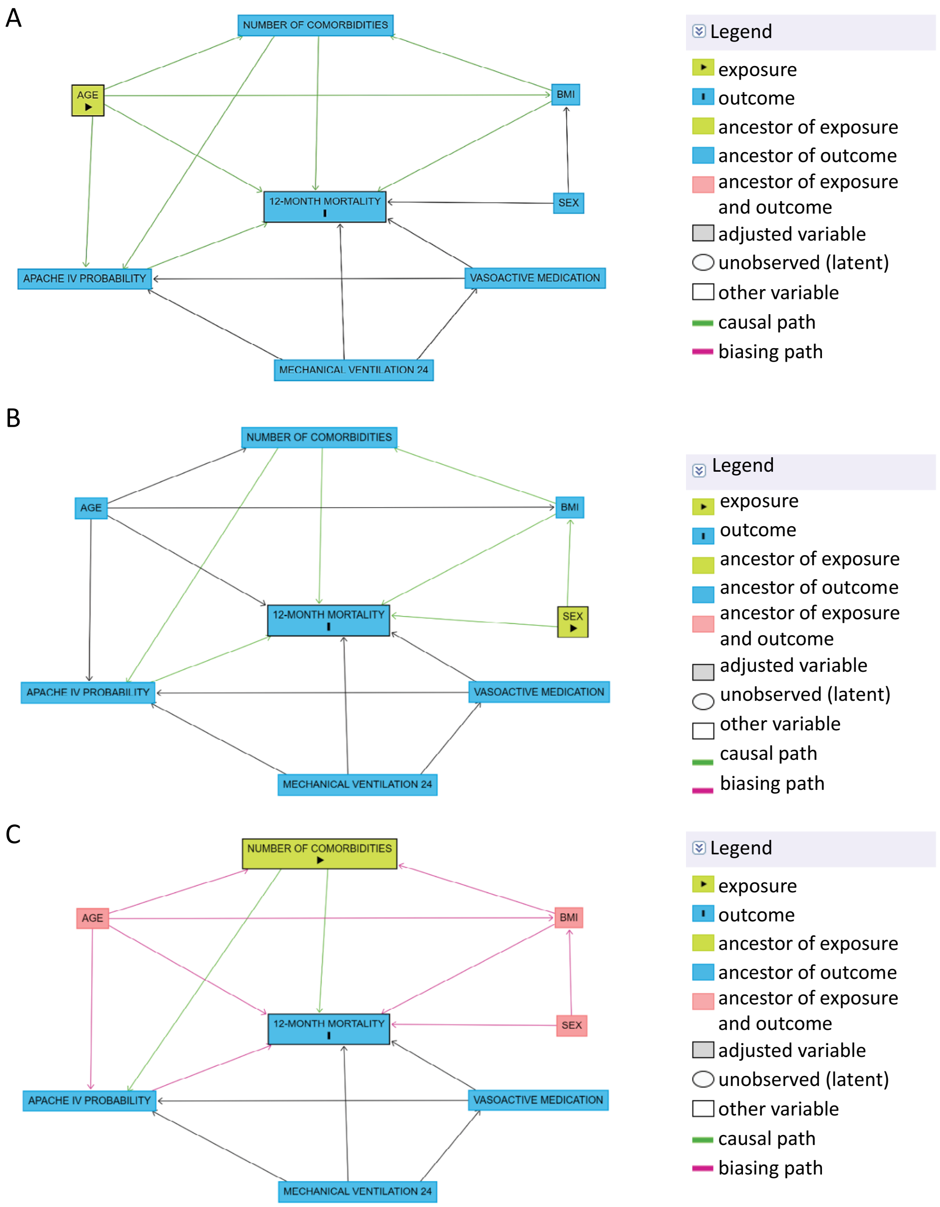

Continue...

This is an open access article under the CC BY license (https://creativecommons.org/licenses/by/4.0/).

...continuation

D

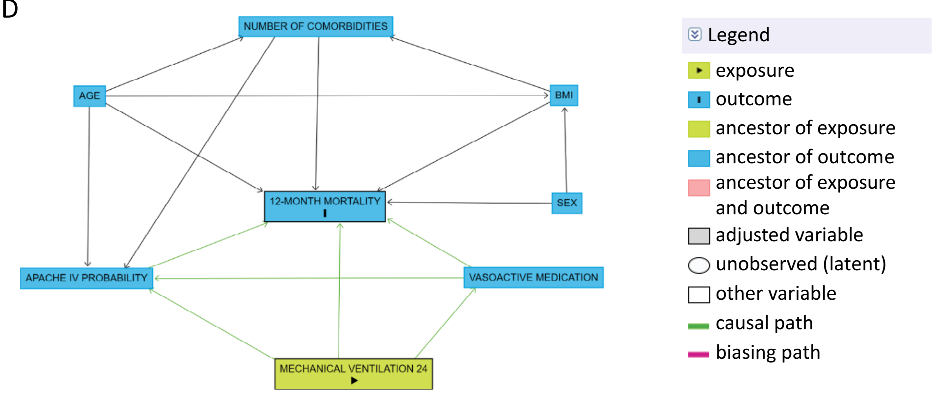

E

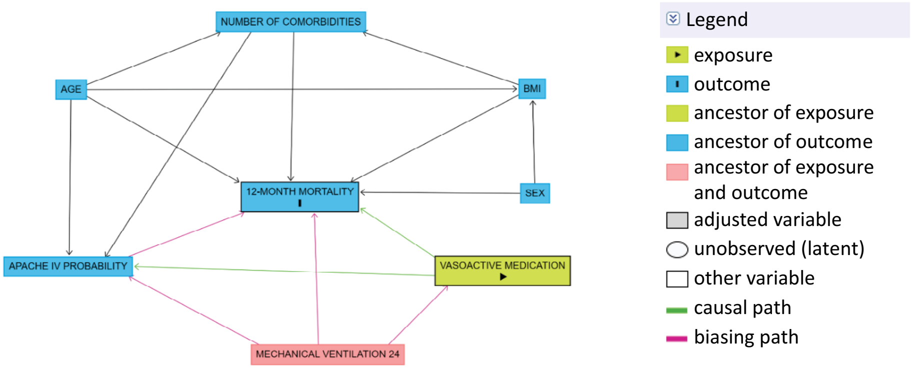

Figure 1S - Directed acyclic graph.

(A) With exposure variable age. Confounders: none; (B) with exposure variable sex. Confounders: none; (C) with exposure variable number of comorbidities. Confounders: age, body mass index; (D) with exposure variable mechanical ventilation in the first 24 hours of intensive care unit admission. Confounders: none; (E) with exposure variable vasoactive medication use in the first 24 hours of intensive care unit admission. Confounders: mechanical ventilation. BMI - body mass index; APACHE - Acute Physiology and Chronic Health Evaluation.

Table 1S - Unadjusted and adjusted hazard ratios for mortality of COVID-19 patients after hospital discharge with adjustment for APACHE IV mortality probability

	Number of patients	HR	95%CI	HRadj	95%CI
Age					
< 40*	942	Reference		Reference	
40-59	4,423	1.53	(0.69 - 3.37)	1.64	(0.73 - 3.66)
60-79	6,166	4.56	(2.15 - 9.69)	4.43	(2.02 - 9.71)
≥ 80	171	21.3	(9.21 - 49.24)	18.36	(7.69 - 43.84)
Sex					
Female*	3,898	Reference		Reference	
Male	7,804	1.35	(1.04 - 1.75)	1.34	(1.03 - 1.73)
Number of comorbidities					
0†	7,383	Reference		Reference	
1	3,228	1.92	(1.47 - 2.5)	1.66	(1.26 - 2.17)
2	763	4.56	(3.31 - 6.28)	3.51	(2.52 - 4.91)
> 2	122	4.67	(2.37 - 9.19)	3.65	(1.82 - 7.29)
MV in first 24 hours of ICU admission					
No*	4,958	Reference		Reference	
Yes	6,744	0.88	(0.7 - 1.11)	0.68	(0.53 - 0.87)
Vasoactive medication in first 24 hours of ICU	6,335	Reference		Reference	
No‡					
Yes	5,367	0.97	(0.77 - 1.23)	0.94	(0.68 - 1.28)

HR - hazard ratio; 95%CI - 95% confidence interval; HRadj - adjusted hazard ratio; MV - mechanical ventilation; ICU - intensive care unit. * Adjustment for APACHE IV mortality probability; † adjustment for APACHE IV mortality probability, age and body mass index; ‡ adjustment for APACHE IV mortality probability and mechanical ventilation.

Table 2S - Characteristics of COVID-19 patients that could be followed-up for 12 months after ICU admission

	Mechanical ventilation	
	**No**	**Yes**
Number of patients	5,062	6869
Age	61 (51 - 69)	61 (53 - 68)
< 40	495 (9.8)	463 (6.7)
40 - 59	1,868 (36.9)	2,624 (38.2)
60 - 79	2,578 (50.9)	3,726 (54.2)
≥ 80	121 (2.4)	56 (0.8)
Sex, male	3,399 (67.1)	4,560 (66.4)
BMI (kg/m^2)	28.3 (25.4 - 32)	29.4 (26.2 - 33.2)
< 18.5	38 (0.8)	29 (0.4)
18.5 - 24.9	1,056 (21.3)	1,081 (16)
25 - 29.9	2,017 (40.7)	2,584 (38.2)
30 - 34.9	1,150 (23.2)	1,846 (27.3)
35 - 39.9	459 (9.3)	795 (11.7)
≥ 40	232 (4.7)	433 (6.4)
Malignancy	105 (2.1)	108 (1.6)
Immunological insufficiency	423 (8.4)	506 (7.4)
Chronic respiratory insufficiency	147 (2.9)	291 (4.2)
Chronic renal failure	156 (3.1)	163 (2.4)
Chronic cardiovascular insufficiency	68 (1.3)	62 (0.9)
Cirrhosis	18 (0.4)	20 (0.3)
Diabetes	1,017 (20.1)	1,400 (20.4)
Number of comorbidities		
0	3,248 (64.2)	4,410 (64.2)
1	1,399 (27.6)	1,952 (28.4)
2	347 (6.9)	448 (6.5)
> 2	68 (1.3)	59 (0.9)
MV in first 24 hours of ICU admission	0 (0)	6,869 (100)
Vasoactive medication in first 24 hours of ICU admission	418 (8.3)	5,057 (73.6)
APACHE III APS	43 (35 - 52)	47 (39 - 57)
APACHE IV mortality probability	0.15 (0.09 - 0.23)	0.22 (0.15 - 0.33)

BMI - body mass index; VM - mechanical ventilation; ICU - intensive care unit; APACHE - Acute Physiology and Chronic Health Evaluation; APS - acute physiology score. Results expressed as n, median (interquartile range) or n (%).

Table 3S - Unadjusted and adjusted hazard ratios for mortality of COVID-19 patients after hospital discharge, excluding patients admitted in the first COVID-19 peak

	Number of patients	HR	95%CI	HRadj	95%CI
Age					
< 40*	870	Reference			
40 - 59	3,808	1.54	(0.7 - 3.4)	-	-
60 - 79	5,220	4.64	(2.18 - 9.87)	-	-
≥ 80	155	20.01	(8.59 - 46.63)	-	-
Sex					
Female*	3,406	Reference			
Male	6,647	1.46	(1.11 - 1.92)	-	-
Number of comorbidities					
0†	6,257	Reference			
1	2,820	1.89	(1.44 - 2.49)	1.62	(1.23 - 2.13)
2	691	4.16	(2.98 - 5.8)	3.36	(2.4 - 4.7)
> 2	114	4.03	(1.97 - 8.26)	3.29	(1.6 - 6.76)
MV in first 24 hours of ICU admission					
No*	4,624	Reference		Reference	
Yes	5,429	0.92	(0.73 - 1.17)	-	-
Vasoactive medication in first 24 hours of ICU admission					
No‡	5,774	Reference		Reference	
Yes	4,279	1.03	(0.81 - 1.31)	1.14	(0.83 - 1.58)

HR - hazard ratio; 95%CI - 95% confidence interval; HRadj - adjusted hazard ratio; MV - mechanical ventilation; ICU - intensive care unit. * No adjustment; † adjustment for age.
