# Supplementary material for: Long-term mortality of Dutch COVID-19 patients admitted to the intensive care medicine: a retrospective analysis from a national quality registry
Source: Crit Care Sci. 2024 Sep 18;36:e202400251en. doi: 10.62675/2965-2774.20240251-en (PMC11463994; doi:10.62675/2965-2774.20240251-en)
Supplement: Translation

# Translation: Mortalidade em longo prazo de pacientes holandeses com COVID-19 internados em terapia intensiva: análise retrospectiva de um registro nacional de qualidade

**DOI:** 10.62675/2965-2774.20240251-pt

**Authors:** Safira A. Wortel, Ferishta Bakhshi-Raiez, Ameen Abu-Hanna, Dave A. Dongelmans, Nicolette F. de Keizer, the Dutch COVID-19 Research Consortium, Aletta Houwink, Allard Dijkhuizen, Annelies Draisma, Annemiek Rijkeboer, Arjan Cloïn, Arthur de Meijer, Auke Reidinga, Barbara Festen-Spanjer, Bas van Bussel, Bob Eikemans, Cretièn Jacobs, David Moolenaar, Dharmanand Ramnarain, Dick Koning, Dirk Boer, Dirk Verbiest, Eline van Slobbe-Bijlsma, Ellen van Koppen, Els Rengers, Erik van Driel, Eva Verweij, Freya van Iersel, Gert Brunnekreef, Hans Kieft, Herman Kreeftenberg, Ilanit Hené, Inge Janssen, Ionana Drogt, Iwan van der Horst, Jan Jaap Spijkstra, Jan Rozendaal, Jannet Mehagnoul-Schipper, Jelle Epker Erasmus, Jessica Holtkamp, Johan Lutisan, Jos van Oers, Judith Lens, Laura van Gulik, Lettie van den Berg, Louise Urlings-Strop, Lyuba Georgieva, Maarten van Lieshout, Marga Hoogendoorn, Marissa Vrolijk-de Mos, Mart de Graaff, Martha de Bruin, Martijn Hoeksema, Martijn van Tellingen, Michel Barnas, Michiel Erkamp, Niels Gritters, Nuray Kusadasi, Paul Elbers, Peter Koetsier, Peter Spronk, Peter van der Voort, Ralph Pruijsten, Remko de Jong, Robert-Jan Bosman, Ronald Wesselink, Ronny Schnabel, Roy van den Berg, Ruud de Waal, Sesmu Arbous, Silvia Knape, Stefaan Hendriks, Tim Frenzel, Tom Dormans, Tom Rijpstra, Vera Silderhuis, Wouter de Ruijter

**Affiliations:** 1 Amsterdã The Netherlands Departamento de Informática Médica, Amsterdam University Medical Center, University of Amsterdam - Amsterdã, The Netherlands.; 2 Amsterdã The Netherlands National Intensive Care Evaluation (NICE) Foundation - Amsterdã, The Netherlands.; 3 Amsterdam The Netherlands Departamento de Medicina Intensiva, Amsterdam University Medical Center, University of Amsterdam - Amsterdam, The Netherlands.; 4 Amsterdã The Netherlands Departamento de Informática Médica, The Dutch COVID-19 Research Consortium - Amsterdã, The Netherlands.; 5 Rijnstate Arnhem The Netherlands Rijnstate - Arnhem, The Netherlands,; 6 Groene Hart Ziekenhuis Gouda The Netherlands Groene Hart Ziekenhuis - Gouda, The Netherlands,; 7 Flevoziekenhuis Almere The Netherlands Flevoziekenhuis - Almere, The Netherlands,; 8 Laurentius Ziekenhuis Roermond Roemond The Netherlands Laurentius Ziekenhuis Roermond - Roemond, The Netherlands,; 9 Saxenburgh Medisch Centrum Hardenberg The Netherlands Saxenburgh Medisch Centrum - Hardenberg, The Netherlands,; 10 Martini Ziekenhuis Groningen The Netherlands Martini Ziekenhuis - Groningen, The Netherlands,; 11 Ziekenhuis Gelderse Vallei Ede The Netherlands Ziekenhuis Gelderse Vallei - Ede, The Netherlands,; 12 Maastricht University Medical Center Maastricht The Netherlands Maastricht University Medical Center - Maastricht, The Netherlands,; 13 Het Van Weel-Bethesda Ziekenhuis Dirksland The Netherlands Het Van Weel-Bethesda Ziekenhuis - Dirksland, The Netherlands,; 14 Elkerliek Ziekenhuis Helmond The Netherlands Elkerliek Ziekenhuis - Helmond, The Netherlands,; 15 Martini Ziekenhuis Groningen The Netherlands Martini Ziekenhuis - Groningen, The Netherlands,; 16 Elisabeth-TweeSteden Ziekenhuis Tilburg The Netherlands Elisabeth-TweeSteden Ziekenhuis - Tilburg, The Netherlands,; 17 Catharina Ziekenhuis Eindhoven The Netherlands Catharina Ziekenhuis - Eindhoven, The Netherlands,; 18 Maasstad Ziekenhuis Rotterdam The Netherlands Maasstad Ziekenhuis - Rotterdam, The Netherlands,; 19 Admiraal De Ruyter Ziekenhuis Goes The Netherlands Admiraal De Ruyter Ziekenhuis - Goes, The Netherlands,; 20 Tergooi MC Hiversum The Netherlands Tergooi MC - Hiversum, The Netherlands,; 21 Haaglanden Medisch Centrum Den Haag The Netherlands Haaglanden Medisch Centrum - Den Haag, The Netherlands,; 22 Canisius Wilhelmina Ziekenhuis Nijmegen The Netherlands Canisius Wilhelmina Ziekenhuis - Nijmegen, The Netherlands,; 23 Alrijne Ziekenhuis Leiden The Netherlands Alrijne Ziekenhuis - Leiden, The Netherlands,; 24 Bernhoven Uden The Netherlands Bernhoven - Uden, The Netherlands,; 25 Bravis Ziekenhuis Roosendaal The Netherlands Bravis Ziekenhuis - Roosendaal, The Netherlands,; 26 ZGT Hengelo The Netherlands ZGT - Hengelo, The Netherlands,; 27 Isala Zwolle The Netherlands Isala - Zwolle, The Netherlands,; 28 St. Anna Ziekenhuis Eindhoven The Netherlands St. Anna Ziekenhuis - Eindhoven, The Netherlands,; 29 Rode Kruis Ziekenhuis Beverwijk The Netherlands Rode Kruis Ziekenhuis - Beverwijk, The Netherlands,; 30 Maasziekenhuis Pantein Beugen The Netherlands Maasziekenhuis Pantein - Beugen, The Netherlands,; 31 Ziekenhuis Nij Smellinghe Drachten The Netherlands Ziekenhuis Nij Smellinghe - Drachten, The Netherlands,; 32 Maastricht University Medical Center Maastricht The Netherlands Maastricht University Medical Center - Maastricht, The Netherlands,; 33 Amsterdam University Medical Centers Amsterdam The Netherlands Amsterdam University Medical Centers - Amsterdam, The Netherlands,; 34 Jeroen Bosch Ziekenhuis s-Hertogenbosch The Netherlands Jeroen Bosch Ziekenhuis - s-Hertogenbosch, The Netherlands,; 35 Viecuri Medisch Centrum Venlo The Netherlands Viecuri Medisch Centrum - Venlo, The Netherlands,; 36 University Medical Center Rotterdam The Netherlands University Medical Center - Rotterdam, The Netherlands,; 37 St. Jans Gasthuis Weert Weert The Netherlands St. Jans Gasthuis Weert - Weert, The Netherlands,; 38 Wilhelmina Ziekenhuis Assen Assen The Netherlands Wilhelmina Ziekenhuis Assen - Assen, The Netherlands,; 39 ZorgSaam Ziekenhuis Terneuzen The Netherlands ZorgSaam Ziekenhuis - Terneuzen, The Netherlands,; 40 IJsselland Ziekenhuis Capelle aan den IJssel The Netherlands IJsselland Ziekenhuis - Capelle aan den IJssel, The Netherlands,; 41 Meander Medisch Centrum Amersfoort The Netherlands Meander Medisch Centrum - Amersfoort, The Netherlands,; 42 HagaZiekenhuis Den Haag The Netherlands HagaZiekenhuis - Den Haag, The Netherlands,; 43 Reinier de Graaf Gasthuis Delft The Netherlands Reinier de Graaf Gasthuis - Delft, The Netherlands,; 44 Beatrixziekenhuis Gorinchem The Netherlands Beatrixziekenhuis - Gorinchem, The Netherlands,; 45 Ziekenhuis Rivierenland Tiel The Netherlands Ziekenhuis Rivierenland - Tiel, The Netherlands,; 46 Isala Zwolle The Netherlands Isala - Zwolle, The Netherlands,; 47 Langeland Ziekenhuis Zoetermeer The Netherlands Langeland Ziekenhuis - Zoetermeer, The Netherlands,; 48 St. Antonius Ziekenhuis Utrecht The Netherlands St. Antonius Ziekenhuis - Utrecht, The Netherlands,; 49 Franciscus Gasthuis & Vlietland Rotterdam The Netherlands Franciscus Gasthuis & Vlietland - Rotterdam, The Netherlands,; 50 Zaans Medisch Centrum Zaandam The Netherlands Zaans Medisch Centrum - Zaandam, The Netherlands,; 51 Ziekenhuis Tjongerschans Heerenveen The Netherlands Ziekenhuis Tjongerschans - Heerenveen, The Netherlands,; 52 Ziekenhuis Amstelland Amstelveen The Netherlands Ziekenhuis Amstelland - Amstelveen, The Netherlands,; 53 Dijklander Ziekenhuis Purmerend The Netherlands Dijklander Ziekenhuis - Purmerend, The Netherlands,; 54 Treant Zorggroep Emmen The Netherlands Treant Zorggroep - Emmen, The Netherlands,; 55 University Medical Center Utrecht Utrecht The Netherlands University Medical Center Utrecht - Utrecht, The Netherlands,; 56 Amsterdam University Medical Centers Amsterdam The Netherlands Amsterdam University Medical Centers - Amsterdam, The Netherlands,; 57 Medisch Centrum Leeuwarden Leeuwarden The Netherlands Medisch Centrum Leeuwarden - Leeuwarden, The Netherlands,; 58 Gelre Ziekenhuizen Apeldoorn The Netherlands Gelre Ziekenhuizen - Apeldoorn, The Netherlands,; 59 University Medical Center Groningen Groningen The Netherlands University Medical Center Groningen - Groningen, The Netherlands,; 60 Ikazia Ziekenhuis Rotterdam The Netherlands Ikazia Ziekenhuis - Rotterdam, The Netherlands,; 61 BovenIJ Amsterdam The Netherlands BovenIJ - Amsterdam, The Netherlands,; 62 OLVG Amsterdam The Netherlands OLVG - Amsterdam, The Netherlands,; 63 St. Antonius Ziekenhuis Utrecht The Netherlands St. Antonius Ziekenhuis - Utrecht, The Netherlands,; 64 Maastricht University Medical Center Maastricht The Netherlands Maastricht University Medical Center - Maastricht, The Netherlands,; 65 Elisabeth-TweeSteden Ziekenhuis Tilburg The Netherlands Elisabeth-TweeSteden Ziekenhuis - Tilburg, The Netherlands,; 66 Amphia Ziekenhuis Breda The Netherlands Amphia Ziekenhuis - Breda, The Netherlands,; 67 Leids Universitair Medisch Centrum Leiden The Netherlands Leids Universitair Medisch Centrum - Leiden, The Netherlands,; 68 Streekziekenhuis Koningin Beatrix Winterswijk Winterswijk The Netherlands Streekziekenhuis Koningin Beatrix Winterswijk - Winterswijk, The Netherlands,; 69 Albert Schweitzer Ziekenhuis Dordrecht The Netherlands Albert Schweitzer Ziekenhuis - Dordrecht, The Netherlands,; 70 Radboud University Medical Center Nijmegen The Netherlands Radboud University Medical Center - Nijmegen, The Netherlands,; 71 Zuyderland Medisch Centrum Heerlen The Netherlands Zuyderland Medisch Centrum - Heerlen, The Netherlands,; 72 Amphia Ziekenhuis Breda The Netherlands Amphia Ziekenhuis - Breda, The Netherlands,; 73 Medisch Spectrum Twente Enschede The Netherlands Medisch Spectrum Twente - Enschede, The Netherlands,; 74 Noordwest Ziekenhuisgroep Alkmaar The Netherlands Noordwest Ziekenhuisgroep - Alkmaar, The Netherlands.

**Keywords:** COVID-19, SARS-CoV-2, Infecções por coronavírus, Tempo de permanência, Alta do paciente, Sobreviventes, Respiração artificial, Mortalidade hospitalar, Avaliação de resultados em cuidados de saúde, Cuidados críticos, Unidades de terapia intensiva, Bases de dados factuais

## Abstract

**Objetivo:**

Descrever a mortalidade em 12 meses de pacientes holandeses com COVID-19 internados em unidades de terapia intensiva, a população total com COVID-19 e vários subgrupos segundo o número de comorbidades, idade, sexo, ventilação mecânica e uso de medicação vasoativa.

**Métodos:**

Incluímos todos os pacientes admitidos com COVID-19 entre 1° de março de 2020 e 29 de março de 2022, do banco de dados do registro holandês *National Intensive Care Evaluation* (NICE). A taxa bruta de mortalidade em 12 meses é apresentada por meio das curvas de sobrevida de Kaplan-Meier para cada subgrupo de pacientes. Usamos modelos de regressão de Cox para analisar os efeitos das características do paciente na mortalidade em 12 meses após a alta hospitalar.

**Resultados:**

Incluímos 16.605 pacientes com COVID-19. A taxa de mortalidade hospitalar foi de 28,1%, e a taxa de mortalidade em 12 meses após a admissão na unidade de terapia intensiva foi de 29,8%. Entre os sobreviventes hospitalares, a mortalidade em 12 meses após a alta hospitalar foi de 2,5% (300/11.931). O risco de morte em 12 meses após a alta hospitalar foi maior em pacientes entre 60 e 79 anos (HR 4,74; IC95% 2,23 - 10,06) e ≥ 80 anos (HR 22,77; IC95% 9,91 - 52,28) do que em pacientes < 40 anos de idade; em pacientes do sexo masculino do que do sexo feminino (HR de 1,38; IC95% 1,07 - 1,78); e em pacientes com uma comorbidade (HR ajustado 1,95; IC95% 1,5 - 2,53), duas comorbidades (HR ajustado 4,49; IC95% 3,27 - 6,16) ou mais de duas (HR ajustado 4,99; IC95% 2,62 - 9,5) do que em pacientes sem comorbidades. Nem o uso de medicação vasoativa nem a ventilação mecânica apresentaram resultados estatisticamente significativos.

**Conclusão:**

A maioria das mortes de pacientes holandeses com COVID-19 na unidade de terapia intensiva ocorreu durante a internação hospitalar. Para os sobreviventes do hospital, a taxa bruta de mortalidade em 12 meses foi baixa. A idade do paciente (acima de 60 anos), o sexo e o número de comorbidades foram associados a um maior risco de morte 12 meses após a alta hospitalar, enquanto a ventilação mecânica e a medicação vasoativa não foram.

## INTRODUÇÃO

Desde o surto da pandemia da doença do coronavírus 2019 (COVID-19), foram publicados muitos estudos sobre a doença, com foco especial nas características dos pacientes, no tratamento e na mortalidade hospitalar. Nos Países Baixos, a taxa média de mortalidade intra-hospitalar de pacientes com COVID-19 em 2020 foi de 31%.^( 1 )^No entanto, foram relatadas diferentes taxas de mortalidade intra-hospitalar entre pacientes com COVID-19 em unidades de terapia intensiva (UTI) em diferentes países, variando de 25% em estudo nacional francês^( 2 )^e 33% em população de estudo alemã^( 3 )^a 59% em pacientes brasileiros de UTI.^( 4 )^Isso pode ser parcialmente explicado por diferenças na organização da assistência médica, pela disponibilidade de leitos de UTI, pela triagem aplicada na admissão na UTI e por políticas de alta hospitalar. Para descartar diferenças nas políticas de alta hospitalar, uma possível solução é analisar a mortalidade em um momento fixo após a alta hospitalar. Embora a mortalidade intra-hospitalar seja importante e forneça informações valiosas, o verdadeiro objetivo dos cuidados na UTI são os desfechos favoráveis do paciente em longo prazo. Em alguns grupos de pacientes em estado crítico, a mortalidade após a alta da UTI ou do hospital é (quase) comparável à da população holandesa em geral, que inclui pacientes cirúrgicos eletivos e cardíacos,^( 5 )^com intoxicação aguda^( 6 )^e relativamente jovens e saudáveis.^( 7 – 9 )^Em alguns outros grupos de pacientes críticos, como pacientes com sepse e pacientes mais velhos com pneumonia, as taxas de mortalidade em longo prazo podem ser substanciais.^( 10 )^Na Suécia, foi relatada taxa de mortalidade em 1 ano de 45% após a admissão na UTI para pacientes com sepse ou choque séptico grave,^( 10 )^enquanto taxas de mortalidade entre 19 e 51%^( 1 , 11 )^foram relatadas em pacientes com pneumonia.

Nosso objetivo consistiu em descrever a mortalidade em longo prazo de pacientes holandeses com COVID-19 internados na UTI. Como as comorbidades preexistentes e outras características do paciente podem influenciar significativamente o desfecho em longo prazo dos pacientes, as análises foram realizadas tanto no grupo total de pacientes com COVID-19 quanto em vários subgrupos com COVID-19 (conforme o número de comorbidades antes da hospitalização, idade, sexo e ventilação mecânica [VM] e o uso de medicação vasoativa nas primeiras 24 horas de admissão na UTI) ajustados para fatores de confusão. Por fim, comparamos a mortalidade em 12 meses da população da UTI com COVID-19 que sobreviveu à internação hospitalar com a mortalidade da população holandesa em geral.

## MÉTODOS

### Fonte de dados

Coletamos dados retrospectivamente do registro holandês *National Intensive Care Evaluation* (NICE). O NICE é um registro nacional de qualidade criado em 1996. Desde 2016, todas as UTIs holandesas participam do NICE e enviam dados regularmente para o registro. Por sua vez, o NICE fornece às UTIs participantes relatórios de avaliação comparativa e acesso a uma ferramenta *on-line* que permite que cada UTI analise seus próprios dados para verificar se é possível aprimorá-los.^( 12 , 13 )^O banco de dados do registro NICE contém, entre outros, dados demográficos, fisiológicos, clínicos, mortalidade na UTI e intra-hospitalar de todos os pacientes com COVID-19 admitidos em UTIs holandesas.^( 12 , 13 )^Considerou-se que os pacientes tinham COVID-19 se a reação em cadeia da polimerase via transcriptase reversa (RT-PCR *- reverse transcription polymerase chain reaction* ) de suas secreções respiratórias fosse positiva para o coronavírus da síndrome respiratória aguda grave 2 (SARS-CoV-2) ou se sua tomografia computadorizada fosse consistente com a COVID-19, ou seja, se o escore do *COVID-19 Reporting and Data System* (CO-RADS) fosse ≥ 4 em combinação com a ausência de um diagnóstico alternativo.^( 14 )^A data da morte após a alta hospitalar foi derivada para cada paciente vinculando os dados do NICE aos do Vektis, um banco de dados nacional de reivindicações administrativas de operadoras de convênio médico.^( 15 )^

### Desfechos do estudo

Incluímos todos os pacientes admitidos com COVID-19 entre 1° de março de 2020 e 29 de março de 2022 do NICE dos Países Baixos. Usamos dois pontos de partida para determinar o período de seguimento: o primeiro foi a data de admissão na UTI e o segundo foi a data de alta hospitalar. Os dados do Vektis permitiram determinar a mortalidade até 12 meses após a admissão na UTI e após a alta hospitalar.

Excluíram-se das análises pacientes cirúrgicos e aqueles com dados importantes ausentes, como data de alta hospitalar e situação da alta hospitalar. Os pacientes com falta de dados sobre o índice de massa corporal (IMC) foram excluídos dos modelos que incluíam o IMC como covariável. Por fim, a taxa de mortalidade esperada em 12 meses para a população holandesa em geral foi avaliada usando as taxas de mortalidade específicas por sexo e idade informadas pela instituição governamental holandesa *Statistics Netherlands* , ou seja, "CBS".^( 16 )^

### Análises estatísticas

As características e os desfechos basais dos pacientes são descritos por meio de estatísticas descritivas. As variáveis contínuas são apresentadas em termos de médias e desvios-padrão (DP) ou medianas e intervalos interquartis (IIQ) dependendo de suas distribuições, enquanto as variáveis categóricas são apresentadas como porcentagens. As características basais são relatadas para não sobreviventes e sobreviventes hospitalares. A mortalidade bruta em longo prazo é apresentada e analisada por meio das curvas de sobrevida de Kaplan-Meier e do teste *log-rank* (Mantel Cox) para comparar as curvas. As características dos pacientes e as curvas de Kaplan-Meier se referem à população total do estudo e a vários subgrupos de pacientes. Esses subgrupos baseiam-se na idade dos pacientes, no sexo, no número de comorbidades e no uso de medicamentos vasoativos e de VM nas primeiras 24 horas de admissão na UTI. Os pacientes foram divididos em grupos etários, sendo definidos os seguintes: pacientes com menos de 40 anos, 40 a 59 anos, 60 a 79 anos e 80 anos ou mais. Para classificar os pacientes com COVID-19 conforme o número de comorbidades (nenhuma, uma, duas ou mais comorbidades), foram consideradas as seguintes comorbidades disponíveis no NICE: imunodeficiência, insuficiência renal crônica, insuficiência respiratória crônica, insuficiência cardiovascular crônica, cirrose, malignidade e diabetes. Em relação à categorização dos pacientes por VM, consideramos quaisquer instâncias de VM ocorrida nas primeiras 24 horas de admissão. Não foi possível fazer outra distinção entre VM invasiva e não invasiva. O uso de medicação vasoativa intravenosa inclui inotrópicos positivos, como vasopressina, dopamina, cloridrato de dobutamina, vasopressores e inibidores de fosfodiesterase, nas primeiras 24 horas de admissão na UTI.

Adotamos modelos de regressão de Cox para analisar os efeitos das características dos pacientes que sobreviveram à admissão hospitalar comparativamente à mortalidade nos 12 meses após a alta hospitalar. Em cada modelo multivariado, ajustamos as covariáveis com base em um gráfico cíclico direcionado (DAG - *directed cyclic graph* )^( 17 )^aplicando o conhecimento clínico. Também aplicamos modelos de regressão de Cox com ajuste complementar de probabilidade de mortalidade do *Acute Physiology and Chronic Health Evaluation* (APACHE) IV como análise de sensibilidade devido às associações entre a probabilidade de mortalidade do APACHE IV e vários fatores de confusão e/ou prognósticos. Essa análise de sensibilidade excluiu, desse modo, os pacientes aos quais se aplicavam os critérios de exclusão do APACHE IV.^( 18 )^Esses critérios incluem pacientes com tempo de internação na UTI inferior a 4 horas ou superior a 1 ano; readmitidos; com diagnóstico de admissão ou tipo de admissão ausentes; queimados; transplantados de órgãos; e da unidade de tratamento coronariano ou em recuperação.

De acordo com as definições de dados do NICE, um paciente em recuperação é um paciente admitido na UTI somente sob a responsabilidade do anestesiologista e imediatamente após uma operação, em que a duração do tratamento na UTI não exceda 4 horas. Por fim, calculamos as proporções de homens e mulheres em cada faixa etária. A média ponderada das taxas de mortalidade da população holandesa em geral foi avaliada conforme a proporção de pacientes em cada grupo. Comparamos a taxa de mortalidade média ponderada em 12 meses da população holandesa em geral com a taxa de mortalidade em 12 meses da população holandesa de UTIs com COVID-19.

Todas as análises foram realizadas por meio do programa estatístico R (versão 4.2.0), enquanto os DAGs foram desenhados por meio do DAGitty versão 3.0^( 19 )^(Figuras 1SA - 1SE - Material Suplementar).

O Comitê de Revisão de Ética Médica do *Academic Medical Center Amsterdam* analisou a proposta de pesquisa e dispensou a necessidade de Termo de Consentimento Livre e Esclarecido (número de referência W21_091 # 21.102).

## RESULTADOS

Foram admitidos 17.686 pacientes graves com COVID-19 em uma UTI holandesa de 1° de março de 2020 a 29 de março de 2022. Depois de excluir pacientes cirúrgicos e com dados de alta ausentes ou com dados de alta hospitalar ausentes (6,1%), incluímos 16.605 pacientes em nossas análises. A tabela 1 mostra as características dos pacientes incluídos. A idade mediana na admissão era de 64 anos; 68,4% dos pacientes incluídos eram do sexo masculino, e 61% dos pacientes foram ventilados mecanicamente nas primeiras 24 horas de admissão na UTI. Nas primeiras 24 horas após a admissão na UTI, 49,9% dos pacientes receberam medicação vasoativa. A comorbidade crônica mais prevalente foi o diabetes (22,3%), seguido de imunodeficiência (9,8%) e insuficiência respiratória crônica (4,3%). A mediana da probabilidade de mortalidade APACHE IV foi de 0,22.

**Tabela 1:** Características dos pacientes com COVID-19 passíveis de seguimento durante 12 meses após a admissão na unidade de terapia intensiva

		Todos os pacientes incluídos na UTI	Hospitalizados sobreviventes	Hospitalizados não sobreviventes
Número de pacientes		16.605	11.931	4.674
Idade		64 (55 - 71)	61 (52 - 69)	70 (63 - 74)
	< 40	1.006 (6,1)	958 (8)	48 (1)
	40 - 59	5.154 (31)	4.492 (37,6)	662 (14,2)
	60 - 79	9.979 (60,1)	6.304 (52,8)	3.675 (78,6)
	≥ 80	466 (2,8)	177 (1,5)	289 (6,2)
Sexo, masculino		11.351 (68,4)	7.959 (66,7)	3.392 (72,6)
IMC (kg/m^2^)		28,7 (25,7 - 32,5)	29 (25,9 - 32,8)	28 (25,2 - 31,8)
	< 18,5	97 (0,6)	67 (0,6)	30 (0,7)
	18,5 - 24,9	3.191 (19,6)	2.137 (18,2)	1.054 (23,1)
	25 - 29,9	6.492 (39,8)	4.601 (39,3)	1.891 (41,4)
	30 - 34,9	3.999 (24,5)	2.996 (25,6)	1.003 (21,9)
	35 - 39,9	1.656 (10,2)	1.254 (10,7)	402 (8,8)
	≥ 40	857 (5,3)	665 (5,7)	192 (4,2)
Malignidade		447 (2,7)	213 (1,8)	234 (5)
Imunodeficiência		1.634 (9,8)	929 (7,8)	705 (15,1)
Insuficiência respiratória crônica		712 (4,3)	438 (3,7)	274 (5,9)
Insuficiência renal crônica		707 (4,3)	319 (2,7)	388 (8,3)
Insuficiência cardiovascular crônica		257 (1,5)	130 (1,1)	127 (2,7)
Cirrose		83 (0,5)	38 (0,3)	45 (1)
Diabetese		3.710 (22,3)	2.417 (20,3)	1.293 (27,7)
Número de comorbidades				
	0	9.836 (59,2)	7.658 (64,2)	2.178 (46,6)
	1	4.974 (30)	3.351 (28,1)	1.623 (34,7)
	2	1.470 (8,9)	795 (6,7)	675 (14,4)
	> 2	325 (2)	127 (1,1)	198 (4,2)
VM nas primeiras 24 horas de admissão na UTI		10.128 (61)	6.869 (57,6)	3.259 (69,7)
Medicação vasoativa nas primeiras 24 horas de admissão na UTI		8.282 (49,9)	5.475 (45,9)	2.807 (60,1)
APACHE III APS		48 (39 - 58)	46 (37 - 55)	54 (44 - 67)
Probabilidade de mortalidade do APACHE IV		0,22 (0,14 - 0,34)	0,19 (0,12 - 0,29)	0,32 (0,22 - 0,48)

UTI - unidade de terapia intensiva; IMC - índice de massa corporal; VM - ventilação mecânica; APACHE - *Acute Physiology and Chronic Health Evaluation* ; APS - escore de fisiologia aguda. Resultados expressos por n, mediana (intervalo interquartil) ou n (%).

Em comparação com os hospitalizados não sobreviventes, os hospitalizados sobreviventes eram mais jovens (idade mediana = 61), tinham proporção menor de homens (66,7%) e menos pacientes com comorbidades (idade mediana = 70, 72,6% do sexo masculino; tabela 1 ).

A tabela 2 mostra os desfechos dos pacientes. O tempo mediano de internação na UTI foi de 11 dias, e o tempo mediano de internação hospitalar foi de 20 dias. No geral, 28% dos pacientes da UTI morreram durante a internação hospitalar. Entre os pacientes com COVID-19 que sobreviveram à internação hospitalar, 2,5% morreram dentro de 12 meses após a alta hospitalar ( Tabela 2 ).

**Tabela 2:** Desfechos dos pacientes com COVID-19

	Todos os pacientes incluídos na UTI
Número de pacientes	16.605
Tempo de internação na UTI em dias	11 (6 - 22)
Tempo de internação hospitalar em dias	20 (12 - 32)
Mortalidade na UTI	4.084 (24,6)
Mortalidade hospitalar	4.674 (28,1)
Mortalidade em 12 meses após a admissão na UTI	4.956 (29,8)
Mortalidade em 12 meses após a alta hospitalar *	300 (2,5)

UTI - unidade de terapia intensiva.

* Calculado para os pacientes da unidade de terapia intensiva que sobreviveram à internação hospitalar (n = 11.931). Resultados expressos como n, mediana (intervalo interquartil) ou n (%).

### Mortalidade bruta por todas as causas em 12 meses

As curvas de sobrevivência de Kaplan-Meier para pacientes com COVID-19 na figura 1 mostram que a mortalidade ocorreu principalmente nos primeiros 2 meses após a admissão na UTI. As curvas de sobrevida de Kaplan-Meier revelaram que pacientes com menos de 40 anos tiveram um desfecho mais favorável do que aqueles de outras faixas etárias; pacientes do sexo feminino tiveram desfecho mais favorável do que os pacientes do sexo masculino; pacientes que não foram ventilados mecanicamente tiveram um desfecho mais favorável do que os pacientes que foram ventilados mecanicamente nas primeiras 24 horas de admissão na UTI; pacientes sem comorbidades tiveram um desfecho mais favorável do que os pacientes com uma, duas ou mais comorbidades; e pacientes que receberam medicação vasoativa nas primeiras 24 horas de admissão na UTI tiveram um desfecho mais desfavorável do que os pacientes que não receberam medicação vasoativa ( Figura 1 ).

**Figura 1:** Curvas de sobrevivência Kaplan-Meier de pacientes com COVID-19 após a admissão na unidade de terapia intensiva.

(A) Todos os pacientes com COVID-19 incluídos; (B) por faixa etária; (C) por sexo; (D) por número de comorbidades; (E) por ventilação mecânica nas primeiras 24 horas de admissão na unidade de terapia intensiva; (F) por uso de medicação vasoativa nas primeiras 24 horas de admissão na unidade de terapia intensiva. UTI - unidade de terapia intensiva.

A figura 2 mostra as taxas de mortalidade bruta após a alta hospitalar de pacientes com COVID-19 e da população holandesa em geral. A mortalidade bruta cumulativa dos pacientes com COVID-19 no fim do período de seguimento de 12 meses foi maior do que a média ponderada das taxas de mortalidade específicas por sexo e idade na população holandesa em geral, ou seja, 2,5% e 0,4%, respectivamente.

**Figura 2:** Curvas de sobrevida de Kaplan-Meier de pacientes com COVID-19 após a alta hospitalar em comparação com as da população holandesa em geral. A linha contínua representa a população do estudo na unidade de terapia intensiva com COVID-19, e a linha tracejada representa a população holandesa em geral.

Com base nos *hazard ratios* (HR) não ajustadas, observamos um risco maior de morte durante os 12 meses após a alta hospitalar em pacientes com idade entre 60 e 79 anos (HR 4,74; intervalo de confiança de 95% [IC95%] 2,23 - 10,06) e pacientes com 80 anos ou mais (HR 22,77; IC95% 9,91 - 52,28) em comparação com pacientes com menos de 40 anos. Além disso, em comparação com pacientes do sexo feminino, os pacientes do sexo masculino apresentaram um risco maior de morte 12 meses após a alta hospitalar (HR 1,38; IC95% 1,07 - 1,78). Pacientes com uma comorbidade (HR 1,95; IC95% 1,5 - 2,53), duas comorbidades (HR 4,49; IC95% 3,27 - 6,16) ou mais de duas comorbidades (HR 4,99; IC95% 2,62 - 9,5) tiveram um risco maior de morte durante os 12 meses após a alta hospitalar em comparação com pacientes sem comorbidades ( Tabela 3 ). Os resultados de VM e medicação vasoativa não foram estatisticamente significativos. Somente os DAGs do número de comorbidades e do uso de medicação vasoativa resultaram em um conjunto de fatores de confusão para o qual foi necessário ajuste. Após o ajuste para idade e IMC, o número de comorbidades ainda foi estatisticamente significativo (HR ajustado 1,69; IC95% 1,3 - 2,2; HR ajustado 3,65; IC95% 2,66 - 5,03; HR ajustado 4,16; IC95% 2,18 - 7,94, para 1, 2 e > 2 comorbidades). Nesse modelo, 211 pacientes (1,8% dos sobreviventes do hospital) foram excluídos devido à falta de dados de IMC. Após o ajuste para VM, a medicação vasoativa não produziu resultados estatisticamente significativos (HR ajustado de 1,08; IC95% 0,8 - 1,46).

**Tabela 3:** *Hazard ratios* não ajustados de mortalidade em pacientes com COVID-19 após a alta hospitalar

		Pacientes	HR	IC95%
Idade				
	< 40	958	Referência	
	40 - 59	4.492	1,56	(0,71 - 3,43)
	60 - 79	6.304	4,74	(2,23 - 10,06)
	≥ 80	177	22,77	(9,91 - 52,28)
Sexo				
	Feminino	3.972	Referência	
	Masculino	7.959	1,38	(1,07 - 1,78)
Número de comorbidades				
	0	7.523	Referência	
	1	3.293	1,95	(1,5 - 2,53)
	2	778	4,49	(3,27 - 6,16)
	> 2	126	4,99	(2,62 - 9,5)
VM nas primeiras 24 horas de admissão na UTI				
	Não	5.062	Referência	
	Sim	6.869	0,85	(0,68 - 1,07)
Medicação vasoativa nas primeiras 24 horas de admissão na UTI				
	Não	6.456	Referência	
	Sim	5.475	0,94	(0,75 - 1,18)

HR - *hazard ratio* ; IC95% - intervalo de confiança de 95%; VM - ventilação mecânica; UTI - unidade de terapia intensiva.

Na análise de sensibilidade, incluímos sobreviventes hospitalares para os quais não se aplicavam os critérios de exclusão do APACHE IV (n = 11.702). Os HRs não ajustados foram semelhantes às das análises principais, tanto em tamanho quanto em significância estatística ( Tabela 3 e Tabela 1S - Material Suplementar). Após o ajuste para a probabilidade de mortalidade APACHE IV, encontramos que os HRs para idade, sexo, número de comorbidades e VM estavam significativamente associados à mortalidade em 12 meses após a alta hospitalar (Tabela 1S - Material Suplementar). Para o uso de medicação vasoativa, o HR ajustado ainda não foi significativamente associado à mortalidade em 12 meses.

Surpreendeu-nos a magnitude dos HRs inferiores a 1 nos modelos de Cox para VM, conforme mostrado na tabela 3 e na tabela 1S (Material Suplementar). Isso nos levou a realizar análises complementares. Comparamos pacientes ventilados mecanicamente com não ventilados mecanicamente com COVID-19. As características dos pacientes dos dois grupos eram comparáveis (Tabela 2S - Material Suplementar). Em outra análise, excluímos os pacientes admitidos na UTI durante o primeiro pico da pandemia da COVID-19 (data de admissão na UTI antes de 24 de maio de 2020) porque a oxigenoterapia mudou após esse período. Essa análise adicional mostrou resultados na mesma direção: um HR de 0,92 (IC95% 0,73 - 1,17) (Tabela 3S - Material Suplementar).

## DISCUSSÃO

### Principais achados

A mortalidade em pacientes holandeses com COVID-19 internados na UTI foi alta durante a internação hospitalar (28%) e ocorreu principalmente nos primeiros 2 meses após a internação na UTI e dentro do hospital. Entre os sobreviventes do hospital, a taxa de mortalidade foi relativamente baixa (2,5%) nos primeiros 12 meses após a alta hospitalar. No entanto, quando comparamos os resultados dos sobreviventes hospitalares com os da população em geral, descobrimos que a população de pacientes com COVID-19 apresentou resultados de mortalidade bruta ligeiramente mais desfavoráveis. Entre os pacientes com COVID-19 sobreviventes à internação hospitalar, aqueles que estavam em faixas etárias mais avançadas (entre 60 e 79 anos e 80 anos ou mais) ou tinham uma, duas ou mais de duas comorbidades apresentaram risco significativamente maior de morte durante os 12 meses após a alta hospitalar do que os pacientes com menos de 40 anos e aqueles sem comorbidades. Além disso, os pacientes que foram ventilados mecanicamente nas primeiras 24 horas após a admissão na UTI tiveram redução no risco de morte nos 12 meses após a alta hospitalar, embora somente depois de ajustarmos a gravidade da doença pelo APACHE IV. Não encontramos nenhum efeito estatisticamente significativo do uso de medicamentos vasoativos na mortalidade em 12 meses após a alta hospitalar.

### Comparação com estudos anteriores

Em um estudo recentemente publicado sobre os desfechos em longo prazo de pacientes com COVID-19, Hägglöf et al. relataram que a mortalidade após 90 dias foi surpreendentemente baixa e que isso indicava uma alta probabilidade de sobrevida após a fase aguda da doença.^( 20 )^Eles relataram que os homens tinham risco maior de mortalidade em 1 ano (RR de 1,13; IC95% 1,04 - 1,24). Esse resultado é consistente com nossos achados. Além disso, relatamos uma taxa de mortalidade intra-hospitalar de 28%, em consonância com outros estudos sobre a COVID-19, embora haja grande variação nas taxas de mortalidade intra-hospitalar relatadas nos estudos com pacientes de UTI com COVID-19 (25% - 59%).^( 2 – 4 , 21 )^A variação na mortalidade pode ser explicada pelo período em que os pacientes foram admitidos na UTI, pela cepa do SARS-CoV-2 e pelas taxas de vacinação contra a COVID-19.^( 22 , 23 )^Outros estudos sobre a COVID-19, como o de Ceccato et al., relataram taxa de mortalidade bruta de 35% em 1 ano, e 1% dos pacientes que receberam alta morreram no primeiro ano após a admissão na UTI.^( 24 )^Em um estudo sueco, foi relatada taxa de mortalidade em 90 dias de 26,9% após a admissão na UTI, e o número de mortes entre 90 dias e o fim do seguimento de março a outubro de 2020 foi baixo (n = 11 de 2.354 pacientes incluídos).^( 25 )^Nesse estudo, no entanto, o período de seguimento foi mais curto, e o sexo masculino foi identificado como um fator de risco para mortalidade (RR 1,28; IC95% 1,06 - 1,55). Em nosso estudo, também descobrimos que o sexo estava associado à mortalidade em longo prazo. Novelli et al. relataram taxa de mortalidade geral em 1 ano de 33,6% para pacientes com COVID-19 e taxa de mortalidade de 3,7% para pacientes com COVID-19 que receberam alta hospitalar com vida.^( 26 )^Isso corresponde aos nossos achados: relatamos uma taxa de mortalidade de 2,5% após 12 meses de alta hospitalar. Embora tenham incluído pacientes com pneumonia e síndrome do desconforto respiratório agudo de um único centro que foram admitidos durante o primeiro pico da COVID-19, houve outras semelhanças. A maioria dos pacientes morreu durante a internação hospitalar, e a idade do paciente acima de 65 anos foi um preditor independente de mortalidade 1 ano após a alta. Em um estudo dinamarquês no qual os pacientes foram seguidos por 6 meses, o sexo masculino, a idade e as comorbidades crônicas foram associados a um maior risco de morte.^( 27 )^

As características dos pacientes associadas à mortalidade em longo prazo também estão condizentes com estudos anteriores na população geral da UTI. Atramont et al. e Szakmany et al. relataram que a idade (mais avançada) estava associada à mortalidade em longo prazo.^( 8 , 28 )^Szakmany et al.^( 28 )^também relataram uma associação entre o número de comorbidades e os desfechos em longo prazo. Em uma análise multivariável, Gayat et al. identificaram idade, comorbidades, transfusão de glóbulos vermelhos, tempo de internação na UTI e anormalidades em fatores clínicos comuns no momento da alta da UTI (pressão arterial sistólica baixa, temperatura, proteína total, contagem de plaquetas e contagem de glóbulos brancos) como fatores independentes associados à mortalidade em 1 ano.^( 7 )^No entanto, não foi possível comparar completamente esses resultados com os do nosso estudo, pois não coletamos as mesmas variáveis, e a regressão logística multivariável foi usada para determinar as variáveis associadas à mortalidade em 1 ano.

Achamos surpreendente a magnitude dos HRs modelos de Cox com ventilação mecânica na tabela 3 e na tabela 1S (Material Suplementar). Um HR menor que 1 sugere que os pacientes ventilados mecanicamente têm melhor sobrevida em 12 meses do que os pacientes com COVID-19 que não são ventilados mecanicamente. Isso nos levou a realizar análises adicionais. Quando comparamos pacientes ventilados mecanicamente com pacientes não ventilados mecanicamente com COVID-19, não observamos grandes diferenças nas características dos pacientes entre os dois grupos (Tabela 2S - Material Suplementar). Em outra análise, excluímos os pacientes admitidos na UTI no primeiro pico da COVID-19 (data de admissão na UTI antes de 24 de maio de 2020) porque a oxigenoterapia mudou durante a pandemia da doença. Durante o primeiro pico da pandemia, a maioria dos pacientes com COVID-19 foi comumente tratada com VM, enquanto entre os pacientes com COVID-19 admitidos após o primeiro pico da pandemia da COVID-19, houve maior disponibilidade de oxigênio nasal de alto fluxo (ONAF), bem como experiência indicando que ele poderia ser usado com segurança. Mais pacientes foram tratados com ONAF e, quando necessário, com VM. O ONAF não é considerado uma forma de VM. A análise adicional revelou resultados na mesma direção: uma RR de 0,92 (IC95% 0,73 - 1,17) (Tabela 3S - Material Suplementar). Esse achado, no entanto, não foi estatisticamente significativo.

Nossos resultados revelaram efeito de reforço do APACHE IV porque o HR de VM diminuiu de 0,85 para 0,68 (Tabela 1S - Material Suplementar). Esse achado não é surpreendente, porque os pacientes ventilados mecanicamente são os mais afetados; portanto, ao ajustar a gravidade da doença, não é surpreendente que o efeito se torne ainda mais forte. O que continua surpreendente é porque a VM parece ter efeito protetor em longo prazo. Como em estudos anteriores, os pacientes que necessitavam de VM tiveram desfechos mais desfavoráveis em longo prazo.^( 29 )^Quando a VM não é escassa, o que ocorre principalmente nos Países Baixos, geralmente consideramos a VM como uma característica do paciente que está em estado mais grave do que aquele que não é ventilado mecanicamente e, portanto, tem chance menor de sobrevida. Entretanto, especialmente no início da pandemia, a VM era escassa. Os resultados parecem mostrar que, nessa população, a VM tem efeito protetor sobre os desfechos em longo prazo. Outra explicação pode ser a confusão residual, pois os prestadores de serviços de saúde decidem sobre a VM com base em dados que não estão disponíveis para nós.

Baseando-se nos dados de nosso estudo, podemos presumir que receber a VM é benéfica em longo prazo em comparação com não receber VM. Normalmente, pensamos na variável VM como uma característica do paciente que está em estado mais grave do que aquele que não é ventilado mecanicamente e, portanto, tem menos chances de sobrevida. No entanto, nessa população estudada com COVID-19, os pacientes não receberam VM durante as primeiras 24 horas de admissão na UTI e deveriam ter sido ventilados mecanicamente porque esse tratamento é protetor em longo prazo. Por fim, os pacientes de nosso estudo que não foram ventilados mecanicamente podem ter recebido VM durante o tratamento na UTI após as primeiras 24 horas de internação naquela unidade. Como não sabemos se todos os pacientes receberam VM após as primeiras 24 horas, não foi possível analisar se isso teve um efeito protetor.

### Pontos fortes e limitações

Este estudo é um dos poucos a relatar um seguimento em longo prazo de 12 meses de pacientes com COVID-19 após o tratamento na UTI. Além disso, este estudo incluiu dados de alta qualidade de um registro nacional de qualidade que abrange todos os pacientes de UTI com COVID-19 em um país inteiro. Este estudo também tem várias limitações. Em comparação com outros países ocidentais, os Países Baixos têm relativamente poucos leitos de UTI; isso pode dificultar a generalização de nossos resultados para outros países ocidentais com maior disponibilidade de UTI. Em comparação com outros contextos, como os países de baixa e média renda (PBMRs), os resultados podem ser difíceis de extrapolar. Em um estudo anterior realizado no Brasil, os pacientes com COVID-19 que necessitavam de VM tiveram sobrevida e qualidade de vida piores.^( 29 )^Além disso, os PBMRs, como o Brasil, têm mais variação nas proporções de pessoal e outros fatores organizacionais do que os Países Baixos.^( 30 )^Felizmente, existem iniciativas como a *Linking of Global Intensive Care* (LOGIC), da qual participam registros nacionais de qualidade e são implementadas ideias de pesquisa para comparar o desempenho das UTIs entre vários países.^( 31 )^

Usamos o banco de dados nacional de reivindicações administrativas Vektis na data do óbito para análises de sobrevida em longo prazo. Se um paciente não tivesse uma data de óbito, esse paciente seria considerado vivo no fim do período do estudo. Isso poderia ter levado a uma superestimação dos sobreviventes. No entanto, a data do óbito foi administrada de maneira oportuna e confiável para interromper diretamente a cobrança de prêmios mensais de convênios médicos de pessoas falecidas; portanto, acreditamos que isso provavelmente não afetou muito os resultados.

### Implicações clínicas e recomendações para estudos futuros

Neste estudo, observamos que a maioria dos pacientes morreu nos primeiros 2 meses após a admissão na UTI e que, após a alta hospitalar, o número de eventos foi baixo. No entanto, os pacientes com COVID-19 ainda tiveram resultados ligeiramente piores do que a população holandesa em geral. Observamos um pequeno aumento na mortalidade imediatamente após a alta hospitalar. Isso pode indicar que os pacientes foram liberados para cuidados domiciliares de fim de vida ou receberam alta para uma unidade de cuidados de fim de vida. Além disso, neste estudo, nos concentramos na sobrevida em longo prazo dos pacientes com COVID-19, contudo, para obter uma compreensão completa das consequências em longo prazo da COVID-19, também é importante analisar resultados em longo prazo mais centrados no paciente, como qualidade de vida e outros desfechos relatados pelo paciente. Estudos menores mostraram que, entre os pacientes com COVID-19, a COVID-19 longa é um problema importante e desafiador.^( 32 – 35 )^

## CONCLUSÃO

Entre os pacientes da unidade de terapia intensiva com COVID-19, a taxa de mortalidade é mais alta nos primeiros 2 meses após a admissão na unidade de terapia intensiva e, depois, estabiliza-se com o tempo. Entretanto, a mortalidade bruta 12 meses após a alta hospitalar é maior do que a da população holandesa em geral. Entre os subgrupos, pacientes com mais de 60 anos, do sexo masculino e com comorbidades apresentaram taxas de risco de morte significativamente mais altas 12 meses após a alta hospitalar do que pacientes com menos de 40 anos, do sexo feminino e sem comorbidades.
